# Novel Variation and Evolution of *AvrPiz-t* of *Magnaporthe oryzae* in Field Isolates

**DOI:** 10.3389/fgene.2020.00746

**Published:** 2020-08-28

**Authors:** Qun Wang, Jinbin Li, Lin Lu, Chengxing He, Chengyun Li

**Affiliations:** ^1^Agricultural Environment and Resource Research Institute, Yunnan Academy of Agricultural Sciences, Kunming, China; ^2^Flower Research Institute, Yunnan Academy of Agricultural Sciences, Kunming, China; ^3^The Ministry of Education Key Laboratory for Agricultural Biodiversity and Pest Management, Yunnan Agricultural University, Kunming, China

**Keywords:** *Magnaporthe oryzae*, pathogenicity, *AvrPiz-t*, haplotype diversity, Rice-*Magnaporthe* interaction

## Abstract

The product of the avirulence (*Avr*) gene of *Magnaporthe oryzae* can be detected by the product of the corresponding resistance (*R*) gene of rice and activates immunity to rice mediated by the *R* gene. The high degree of variability of *M. oryzae* isolates in pathogenicity makes the control of rice blast difficult. That resistance of the *R* gene in rice has been lost has been ascribed to the instability of the *Avr* gene in *M. oryzae*. Further study on the variation of the *Avr* genes in *M. oryze* field isolates may yield valuable information on the durable and effective deployment of *R* genes in rice production areas. *AvrPiz-t* and *Piz-t* are a pair of valuable genes in the Rice-*Magnaporthe* pathosystem. *AvrPiz-t* is detectable by *Piz-t* and determines the effectiveness of *Piz-t*. To effectively deploy the *R* gene *Piz-t*, the distribution, variation, and evolution of the corresponding *Avr* gene *AvrPiz-t* were found among 312 *M. oryzae* isolates collected from Yunnan rice production areas of China. PCR amplification and pathogenicity assays of *AvrPiz-t* showed that 202 isolates (64.7%) held *AvrPiz-t* alleles and were avirulent to IRBLzt-T (holding *Piz-t*). There were 42.3–83.3% avirulent isolates containing *AvrPiz-t* among seven regions in Yunnan Province. Meanwhile, 11 haplotypes of *AvrPiz-t* encoding three novel *AvrPiz-t* variants were identified among 100 isolates. A 198 bps insertion homologous to solo-LTR of the retrotransposon inago2 in the promoter region of *AvrPiz-t* in one isolate and a frameshift mutation of CDS in another isolate were identified among 100 isolates, and those two isolates had evolved to virulent from avirulent. Synonymous mutation and non-AUG-initiated N-terminal extensions keeps the *AvrPiz-t* gene avirulence function in *M. oryzae* field isolates in Yunnan. A haplotype network showed that H3 was an ancestral haplotype. Structure variance for absence (28.2%) or partial fragment loss (71.8%) of *AvrPiz-t* was found among 39 virulent isolates and may cause the *AvrPiz-t* avirulence function to be lost. Overall, *AvrPiz-t* evolved to virulent from avirulent forms via point mutation, retrotransposon, shift mutation, and structure variance under field conditions.

## Introduction

During the long history of coevolution between plants and pathogens, two layers of host immunity have developed in plants (Jones and Dangl, 2006). One is PAMP-triggered immunity (PTI), the other is effector-triggered immunity (ETI). PTI usually recognizes the conserved pathogen PAMPs by plant cell surface pattern recognition receptors (PRRs), and then activates the relatively weak basic defense response in plants. PAMPs are common features of microbial pathogens, such as bacterial flagellin or fungal chitin, and lipopolysaccharides. ETI is triggered when plant resistance proteins (R proteins) directly or indirectly recognize the effector proteins secreted by pathogens, and then stimulate a stronger resistance response to inhibit the infection of pathogens (Chisholm et al., 2006; Dodds and Rathjen, 2010). Effectors are divided into virulent and avirulent effectors depending on its pathogenicity to the host. Avirulence (Avr) proteins can interact directly or indirectly with the corresponding resistance (*R*) protein or combine to the promoter of the corresponding *R* genes, then trigger the ETI. Race-specific plant *R* genes have the ability to detect the corresponding Avr protein and induce effective resistance to plant pathogens (Dai et al., 2010). Specific recognition and interaction between *Avr* and *R* genes is the key to induce plant resistance. Through mutation or complete loss of effector genes, the pathogens are able to evade recognition of R proteins, infect plants, and counter efforts to control pivotal plant diseases (McDonald and Linde, 2002; Singh et al., 2011). Further analysis of *Avr* genes of the field isolate in a plant pathogen may provide precious information for the utilization of *R* genes in field crops (Stukenbrock and McDonald, 2009).

Rice blast is one of the most destructive diseases in rice worldwide (Dean et al., 2012; Nalley et al., 2016; Deng et al., 2017). It is caused by the fungus *Magnaporthe oryzae*. The amount of rice lost annually to this fungal disease is enough to feed more than 60 million people (Choi et al., 2013). The deployment of rice resistant cultivars containing major resistance gene (*R*) is the most economical, effective, and environmentally friendly measure to control rice blast disease. The high degree of variability of *M. oryzae* isolates in pathogenicity makes the control of rice blast difficult (Sharma et al., 2002). That resistance of the *R* gene in rice has been lost has been ascribed to the instability of *Avr* in *M. oryzae* (Khang et al., 2008). To date, there are more than thirty rice blast *R* genes and twelve *Avr* genes to be cloned (Wang B. H. et al., 2017). Mostly, *R* genes in rice encode NLR proteins, and most cloned *Avr* genes in *M. oryzae* encode small secreted proteins. These cloned *Avr* genes include *PWL1* (Kang et al., 1995), *PWL2* (Sweigard et al., 1995), *AVR1-CO39* (Farman and Leong, 1998), *AVR-Pita* (Orbach et al., 2000), *ACE1* (Fudal et al., 2005), *AvrPiz-t* (Li et al., 2009), *Avr-Pia* (Yoshida et al., 2009), *Avr-Pii* (Yoshida et al., 2009), *Avr-Pik/km/kp* (Yoshida et al., 2009), *AvrPib* (Zhang et al., 2015), *AvrPi9* (Wu et al., 2015), and *AvrPi54* (Ray et al., 2016), which play a critical role in clarifying the genes’ variance and the interaction with correspondent *R* genes in the Rice-*Magnaporthe* pathosystem (Wang B. H. et al., 2017).

Among the cloned *Avr* genes of *M. oryzae*, *AvrPiz-t* activates immunity to rice mediated by the *R* gene *Piz-t* in a gene-for-gene theory (Luo et al., 2005; Li et al., 2009). *AvrPiz-t* was cloned from the isolate 81278ZB15. It is comprised of an intronless gene in chr7 and encodes a small predicted secreted protein consisting of 108 amino acids with no homology to known proteins (Li et al., 2009; Park et al., 2012). The 18-aa N-terminal secretion signal at the amino-terminus is critical for the avirulent function of *AvrPiz-t*. A transposon Pot3 insertion and a base substitution have resulted in the avirulence function of *AvrPiz-t* gene being lost (Li et al., 2009). The corresponding resistant gene *Piz-t* is a broad spectrum NLR protein gene (Hayashi et al., 2004). The broad spectrum resistant gene has been cloned from the rice cultivar Toride1 (Zhou et al., 2006). The successful cloning of the pair *Avr*/*R* genes facilitates their interaction study. The Avr effector AvrPiz-t preferentially gathers in the biotrophic interfacial complex (BIC), and then is transferred into rice cells (Park et al., 2012). It can suppress PTI because ectopic expression of *AvrPiz-t* inhibits the generation of reactive oxygen species (ROS) induced by the flg22 and chitin in transgenic rice and invigorates susceptibility to *M. oryzae*. Recent studies have shown that *Piz-t* interacts indirectly with *AvrPiz-t* (Park et al., 2012, 2016; Wang R. et al., 2016; Tang et al., 2017). Effector AvrPiz-t participates in both PTI and ETI of rice to *M. oryzae*. 12 APIPs (AvrPiz-t interacting proteins) have been identified as targets of AvrPiz-t. AvrPiz-t interacting with four APIPs has been discovered. APIP6 is a functional ring E3 ubiquitin ligase. AvrPiz-t both interacts with APIP6 and reduces their E3 ligase activity by promoting their degradation. In response, APIP6 can ubiquitinate and degrade AvrPiz-t (Park et al., 2012). The interaction of APIP6 and AvrPiz-t plays a role in suppressing the PAMP-triggered immunity (PTI) of rice. APIP10 is another E3 ligase. Except for ubiquitinating and degrading AvrPiz-t, it is also a negative regulator that accumulates and activates the NLR receptor Piz-t (Park et al., 2016). APIP10 functionally connects AvrPiz-t to its receptor Piz-t in rice. Transcription factors (TFs) play an important role in regulating and activating NLR proteins in plants (Chang et al., 2013; Inoue et al., 2013; Xu et al., 2014). As a bZip TF, APIP5 both interacts with effector AvrPiz-t from M. oryzae and the NLR protein Piz-t from rice (Wang R. et al., 2016). ETN triggered by AvrPiz-t is suppressed by NLR receptor Piz-t. And rice utilizes APIP5 as a decoy substrate of AvrPiz-t for preventing ETN at the necrotrophic stage against *M. oryzae*. APIP5 negatively regulates the cell death binding to AvrPiz-t and its transactivation activity and protein accumulation are suppressed at the necrotrophic stage. Piz-t suppresses AvrPiz-t-mediated APIP5 turnover by interacting with and stabilizing the protein to prevent host necrosis. At the same time, APIP5 plays a key role in Piz-t stability during infection. As a nucleoporin2 domain containing protein, APIP12 is a virulence target of AvrPiz-t and is involved in the basal resistance against *M. oryzae* in rice (Tang et al., 2017). It finishes interactions with both AvrPiz-t and APIP6 through different regions of the proteins.

Significant progress has been made in the indirect interaction of *AvrPiz-t* and its cognate resistance gene *Piz-t* has. According to gene-for-gene theory, the host is resistant to the pathogen if corresponding *R* and *Avr* genes exist in both host and pathogen. The host is susceptible if either is absent or inactive (Flor, 1971). So, variance or the presence/absence of *AvrPiz-t* gene of *M. oryzae* field isolates determines the stability and effectiveness of corresponding *R* gene *Piz-t*. The integral identification of resistance in the national rice blast garden of China in the 1970s showed that the *Piz-t* gene contained higher resistance to *M. oryzae* (Ling et al., 1986). *Piz-t* has kept its higher resistance to rice blast in some provinces of China, such as Yunnan, Jilin, Jiangsu, Zhejiang Province (Zhou et al., 2003), and Guangdong Province (Zhu et al., 2004; Yang et al., 2008). Lower virulent frequency (8.46%) of *M. oryzae* to *Piz-t* was found in the Northern G/J rice region of China (Lei et al., 2000). 80% *M. oryzae* isolates in the Yunnan Province of China were avirulent to *Piz-t* (Li et al., 2011). Lower virulent frequency of *AvrPiz-t* and higher resistant frequency of *Piz-t* suggests that *AvrPiz-t* and *Piz-t* are a pair of valuable genes in the Rice-*Magnaporthe* pathosystem in some provinces of China. Sequence variance on open reading frames (ORFs) and size variance on promoter regions of *AvrPiz-t* have been detected from 38 countries and regions (Chen et al., 2014). Yunnan Province of China holds abundant resources of both rice and blast fungus (Jiang, 1995). Variance and evolution of the *AvrPiz-t* gene in *M. oryzae* isolates have not been reported. In this paper, we report the molecular mechanisms of variance and evolution of *AvrPiz-t* in *M. oryzae* field isolates in Yunnan Province of China. This will be helpful to evaluate the resistant efficiency and durability of *Piz-t*.

## Materials and Methods

### Fungal Isolates, Rice Cultivars, Culture, and Pathogenicity Assays

A total of 312 single-spore purified isolates were obtained from 14 prefectures of seven regions in Yunnan Province (Supplementary Table S1). These isolates are from four prefectures (KM, YX, CX, and QJ) of central Yunnan, three prefectures (DL, BS, and DH) of Western Yunnan, one prefecture (LJ) of Northwestern Yunnan, one prefecture (ZT) of Northeastern Yunnan, two prefectures (HH and BN) of Southern Yunnan, two prefectures (LC and PE) of Southwestern and one prefecture (WS) of Southeastern Yunnan. According to the production area, these isolates were divided into *GJ* from *Geng/Japonica* rice area or *XI* from *Xian/Indica* rice area (Supplementary Table S1). Rice monogenic line IRBLzt-T containing the *Piz-t* and the susceptible control variety, Lijiangxintuanheigu (LTH) without *Piz-t*, were used for pathogenicity assays. In a previous study (Li et al., 2014), methods of isolate storage, spore cultivation, rice seedlings culture, and the pathogenicity assay were described. Disease reactions were judged by a modified pathogenicity assay (Jia et al., 2003). Seven days after inoculation, the disease reactions were evaluated according to lesions’ visual number and amount at the second youngest leaf. The pathogenicity assay was repeated three times.

### DNA Preparation, PCR Amplification, and DNA Sequencing

Fungal isolates were cultured at 25°C under dark conditions in complete liquid media to produce mycelium over 6 to 8 days. Using the CTAB method (Tai and Tanksley, 1990), DNA was extracted from mycelia. Forward Primer AvztF and reverse primer AvztR, published in a previous study (Li et al., 2009), were specifically applied to amplify complete genes, including non-CDS and CDS. Each PCR reaction were comprised of the following components: 25 μl of 2 × Pfu PCR MasterMix (Tiangen Biotech Co., LTD, Beijing, China), 1 μl of each 10 μM primer, 2 μl of fungal genomic DNA, and 21 μl distilled water (provided by Tiangen Kit). PCR amplification was implemented in a BIO-RAD Thermal Cycler (C1000, Bio-Rad Laboratories, Life Science Research, Hercules, CA, United States). The PCR program is as follows: 1 cycle at 95°C for 3 min, followed by 35 cycles at 95°C for 30 s, 55°C for 30 s, 72°C for 30 s, and a final extension of 72°C for 7 min. The size of the PCR product was estimated by DL2000 DNA Ladder (Tiangen Biotech Co., LTD., Beijing, China). Using the PCR amplifying primers and five *AvrPiz-t* internal sequencing primers, such as AvrPizt-R1, AvrPizt-R2, C8-W1F-F01, F3-W1F-F09, and C8-CW1F-B08 (Supplementary Table S2 and Supplementary Figure S1), PCR products were sequenced by Shanghai Life Technologies Biotechnology Co., Ltd (Shanghai, China). All reactions of PCR amplification and sequencing were repeated three times. The selection criteria for isolates sequencing are as follows: (1) Randomly selected isolates may cover the sampled region as far as possible in order to know the gene’s genetic variation as a whole; (2) It is ensured that no less than 3 isolates were sequenced in every sampled prefecture; (3) If isolates with *AvrPiz-t* gene (+) are very few in a prefecture, all will be sequenced; and (4) All of V + isolates are sequenced.

In order to understand whether virulent isolates display the structure variance of *AvrPiz-t* gene’s partial deletion or absence, three pairs of primers (AvrPizt-F1/R1, AvrPizt-F2/R2, and AvrPizt-F3/R3) were designed (Supplementary Table S2 and Supplementary Figure S1) to respectively amplify three fragments (5′, middle, and 3′ fragment) of *AvrPiz-t* gene based on the DNA sequences of *AvrPiz-t* (GenBank accession no. EU837058). Amplification products were detected by 1% agarose electrophoresis, the desired band was marked with “+,” no band with “−”. According to the electrophoresis results, every virulent isolate was given a genotype code of either “−−−” (no amplifications with three fragments), “+ + −” (no amplification with 3′ fragment), “+ − +” (no amplification with middle fragment), “− + +” (no amplification with 5′ fragment), “+ −−”(only amplification with 5′ fragment), “− + −” (only amplification with middle fragment), “−− +” (only amplification with 3′ fragment), or “+ + +” (amplifications with three fragments). Amplicons were sequenced according to the above methods. Disorder sequences were cloned according to the pClone007 vector Kit (Beijing TsingKe Biotech Co., Ltd.) and sequenced.

### Data Analysis

Sequences were assembled with Seqman software in lasergen7.0 (Burland, 2000). Assembled sequences were aligned with ClustalW 1.81 (Thompson et al., 1997). The number of DNA haplotypes, polymorphic sites, nucleotide diversity (π), and haplotype diversity (*H*_*d*_) (Nei, 1987; Nei and Miller, 1990) were calculated with the program DNA Sequence Polymorphism (Rozas et al., 2003). Neutrality tests of Tajima’s D (Tajima, 1989), Fu, and Li’s D^∗^ (Fu and Li, 1993) were also conducted using this program. A haplotype distribution map was constructed using ArcGIS 10.2 (Redlands, CA, United States), in which prefecture coordinates were represented by the capital coordinates of the prefecture. A haplotype network analysis was run using TCS1.21^1^ (Clement et al., 2000). In order to clarify the possible history of populations, based on the resulting network, a nested clade analysis (NCA) was performed using Geodis 2.5 (Posada et al., 2000).

## Results

### The Frequency and Distribution of Avirulence Gene *AvrPiz-t* in Yunnan Province

From the pathogenicity assay, 271(86.9%) of the 312 tested isolates were avirulent to the IRBLzt-T (containing *Piz-t*), while the remaining 41 isolates were virulent (Table 1). The frequency of avirulence to *Piz-t* was 100, 91.8, 90.5, 90.2, 85.7, 78.3, and 53.8% in northwestern, central, western, northeastern, southeastern, southern, and southwestern prefectures, respectively. By *AvrPiz-t* specific amplification, 204 (65.4%) out of 312 isolates were detected to hold the *AvrPiz-t* alleles, including 202 avirulent and two virulent isolates (Table 1). The detection frequency of *AvrPiz-t* was 83.3, 81.6, 78.4, 59.1, 57.1, 56.5, and 46.1% in northwestern, central, northeastern, western, southeastern, southern, and southwestern prefectures, respectively.

**TABLE 1:** Geographical information, pathogenicity and *AvrPiz-t* gene amplification among *Magnaporthe oryzae* isolates in Yunnan Province of China.

**Location**	**Lat.**	**Long.**	**No.**	**Pathogenicity assay**		***AvrPiz-t* PCR detection**			
				**A (%)**	**V (%)**	** +**		***–***	
						**A (%)**	**V (%)**	**A (%)**	**V (%)**
Central			49	45(91.8)	4(8.2)	40(81.6)	0(0)	5(10.2)	4(8.2)
KM	25.05	102.72	26	22(84.6)	4(15.4)	18(69.2)	0(0)	4(15.4)	4(15.4)
YX	24.35	102.55	8	8(100)	0(0)	7(87.5)	0(0)	1(12.5)	0(0)
CX	25.5	103.8	3	3(100)	0(0)	3(100)	0(0)	0(0)	0(0)
QJ	25.03	101.55	12	12(100)	0(0)	12(100)	0(0)	0(0)	0(0)
Western			137	124(90.5)	13(9.5)	81(59.1)	0(0)	43(31.4)	13(9.5)
DL	25.6	100.23	6	6(100)	0(0)	6(100)	0(0)	0(0)	0(0)
BS	25.12	99.17	83	79(95.2)	4(4.8)	52(62.7)	0(0)	27(32.5)	4(4.8)
DH	24.43	98.58	48	39(81.3)	9(18.7)	23(47.9)	0(0)	16(33.3)	9(18.7)
Northwestern			12	12(100)	0(0)	10(83.3)	0(0)	2(16.7)	0(0)
LJ	26.88	100.23	12	12(100)	0(0)	10(83.3)	0(0)	2(16.7)	0(0)
Northeastern			51	46(90.2)	5(9.8)	40(78.4)	0(0)	6(11.8)	5(9.8)
ZT	27.33	103.72	51	46(90.2)	5(9.8)	40(78.4)	0(0)	6(11.8)	5(9.8)
Southern			23	18(78.3)	5(21.7)	12(52.2)	1(4.3)	6(26.1)	4(17.4)
HH	23.37	103.4	17	15(88.2)	2(11.8)	9(52.9)	0(0)	6(35.3)	2(11.8)
BN	22.02	100.8	6	3(50)	3(50)	3(50)	1(16.7)	0(0)	2(33.3)
Southwestern			26	14(53.8)	12(46.2)	11(42.3)	1(3.8)	3(11.5)	11(42.3)
LC	23.88	100.08	18	9(50)	9(50)	6(33.3)	1(5.6)	3(16.7)	8(44.4)
PE	23.07	101.03	8	5(62.5)	3(37.5)	5(62.5)	0(0)	0(0)	3(37.5)
Southeastern			14	12(85.7)	2(14.3)	8(57.1)	0(0)	4(28.6)	2(14.3)
WS	23.37	104.25	14	12(85.7)	2(14.3)	8(57.1)	0(0)	4(28.6)	2(14.3)
Total			312	271(86.9)	41(13.1)	202(64.7)	2(0.6)	69(22.1)	39(12.5)
GJ			200	187(93.5)	13(6.5)	148(74.0)	0(0)	39(19.5)	13(6.5)
XI			112	84(75.0)	28(25.0)	54(48.2)	2(1.8)	30(26.8)	26(23.2)
Total			312	271(86.9)	41(13.1)	202(64.7)	2(0.6)	69(22.1)	39(12.5)

*No.: isolate numbers in Pathogenicity assay and PCR detection of *AvrPiz-t* gene; A: avirulent (A) isolate numbers in pathogenicity assay, percentage in parentheses; V: virulent (V) isolate numbers in pathogenicity assay, percentage in parentheses; + : isolates with amplification product of *AvrPiz-t* gene; −: isolates without amplification product of *AvrPiz-t* gene numbers in PCR detection of *AvrPiz-t* gene, percentage in parentheses; *GJ* and *XI* indicate isolates from *Geng/Japonica* and *Xian/Indica* rice production area, respectively.*

Combining the results of pathogenicity and amplification, 202 avirulent isolates and 39 virulent isolates complied with the gene-for gene-fashion. According to prefecture level, the percentage of avirulent isolates with *AvrPiz-t* was 64.7%, ranging from 38.9% of LC to 100% of CX, QJ, and DL (Table 1). Five prefectures (CX, QJ, DL, YX, and LJ) were more than 80%, two prefectures (DH and LC, respectively, 47.9 and 38.9%) were lower than 50%. The percentage of avirulent isolates with the *AvrPiz-t* gene changed from 42.3% in the southwestern prefecture to 83.3% of northwestern among seven regions with a rising tendency. These results suggest that these field *M. oryza*e isolates can still be beneficial to its correspondent resistant gene *Piz-t* in managing rice blast disease in Yunnan’s rice growing regions, except for limited use in LC of Southwestern Yunnan. Interestingly, the percentages of avirulent isolates with the *AvrPiz-t* gene in *GJ* was higher than *XI* in Yunnan, respectively, 74 and 48.2%. It suggests that the *Piz-t* gene is more suitable for the *GJ* rice-growing region in Yunnan.

### *AvrPiz-t* Haplotypes Variance and Distribution

**FIGURE 1:**
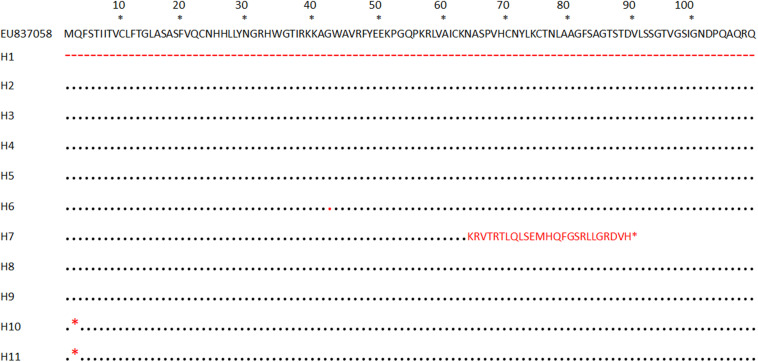
Amino acid alignment of *AvrPiz-t* coding region from eleven haplotypes in Yunnan Province as compared with EU837058 (single letter code is used for amino acids, red remarks representative variant amino acids locus, - means absent, ^∗^ means stop codon).

*AvrPiz-t* gene was successfully amplified from 202 avirulent and two virulent isolates out of 312 isolates when applying specified primers. The remaining 108 isolates (69 avirulent and 39 virulent isolates) had no amplification bands seen by PCR detection. Amplification products of 100 isolates, including 98 avirulent and two virulent isolates, were sequenced. Published *AvrPiz-t* gene sequence EU837058, which is 2507 bp in length, acted as a reference sequence. After assembled and aligned analysis, 10 variable sites were detected (Table 2 and Supplementary Figure S2), in which seven sites (V1 toV5, V9 and V10) were located in non-coding regions (non-CDS) and three sites (V6 to V8) in the coding region (CDS). These variable sites include three indels and seven transitions. In total, 11 haplotypes based on 100 *AvrPiz-t* sequences were identified, including the original haplotype H3 (EU837058, isolate 81278ZB15 *AvrPiz-t* gene) and at least nine novel haplotypes, comprised of 30 Yunnan *M. oryzae* isolates (Table 2). Amino acid sequences of *AvrPiz-t* coding region from eleven haplotypes in Yunnan Province as compared with EU837058 were translated (Figure 1).

**TABLE 2:** Haplotypes of *AvrPiz-t* gene of *M. oryzae* isolates in the field of Yunnan, China.

**Haplotypes**	**No. isolates (%)**	**Variant nucleotide position^*a*^**									
		**Non-CDS**					**CDS**			**Non-CDS**	
		**V1**	**V2**	**V3**	**V4**	**V5**	**V6**	**V7**	**V8**	**V9**	**V10**
				
		**159**	**160**	**560**	**672**	**1387**	**1563**	**1685**	**1751**	**1954**	**2435**
EU837058		C	T	T	A	–	C	T	–	A	T
H1	1(1)	*	*	*	*	insertion^*b*^	*	*	–	*	*
H2	1(1)	*	*	–	*	–	*	*	–	*	*
H3	69(69)	*	*	*	*	–	*	*	–	*	*
H4	1(1)	*	*	–	*	–	*	*	–	*	C
H5	20(20)	*	*	*	*	–	*	*	–	*	C
H6	3(3)	*	*	*	*	–	*	C	–	*	*
H7	1(1)	*	*	*	*	–	*	*	A	G	*
H8	1(1)	*	*	*	G	–	*	*	–	*	*
H9	1(1)	*	C	*	*	–	*	*	–	*	*
H10	1(1)	T	*	*	*	–	T	*	–	*	*
H11	1(1)	*	*	*	*	–	T	*	–	*	*

^*a*^Variable site location in the *AvrPiz-t* gene (EU837058); ^*b*^198 bp insertion, the bases as followed: CACGGCCAGGGTAAGCATAGCACGGAAGCTAGTCTATT GGCGCCTATCGACGAAGATTCTACTCTGAGGAGAAGAAAGGAAACAAACAAAGTTCTGAGCTTGACGCGACGTAGATGAAGGTATCACCCTGGTGGGTCGTTGGCCAGG GGTCAGGGTTAGGGTCTGGTCATCTCATGGTCCAAATGTCACAGCATTTGT. “*” is identical to EU837058.

Among the 11 haplotypes of the *AvrPiz-t* gene, H1 and H7 are virulent haplotypes. Nine haplotypes (H2 to H6 and H8 to H11) are avirulent to IRBLzt-T. A 198-bp insertion at a position 173 bp upstream from the start codon of *AvrPiz-t* was found in H1 represented by a single virulent isolate 07-231-2a in LC (Table 2). The reverse-complement sequence of the 198-bp insertion was homologous (97%) to solo-LTR of retrotransposons Inago2. Inago2, belonging to the Ty3/gypsy family of retrotransposons, was identified from *M. oryzae* Japanese field isolate 9439009 (Sanchez et al., 2011), whose copies were distributed within the genome of *Magnaporthe* spp. (Sanchez et al., 2011). According to pathogenicity results, 07-231-2a is virulent to IRBLzt-T containing *Piz-t* gene (Supplementary Figure S3). The insertion of solo-LTR of retrotransposons Inago2 in promoter regions is predicted to change the avirulent function of the isolate 07-231-2a. Inago2 was founded at 41 bp upstream ORF in Chinese isolates without pathogenicity results (Chen et al., 2014). So, the inserting location and element of inago2 in this study are different from the previous study. An adenine insertion at a position 192 bp from the start codon of *AvrPiz-t* in H7 represented by a single isolate 08-39-2d in BN was observed and shaped a frameshift mutation from the 64th to the 88th codon that introduced twenty-five aberrant amino acids culminating in the stop codon in the 89th codon (Table 2 and Figure 1). According to pathogenicity results, 08-39-2d is virulent to IRBLzt-T containing *Piz-t* gene (Supplementary Figure S3). As a result, the frameshift mutation is predicted to produce truncated proteins and lead the isolate 08-39-2d to be virulent to *Piz-t*. The insertion in H7 is the same as the VII haplotype based on *AvrPiz-t* ORF sequence (Chen et al., 2014). The VII haplotype is determined only by variable sites of ORF sequence; however, haplotypes were determined by variable sites of coding and non-coding region sequences of *AvrPiz-t* gene in this study. Whether H7 is identical to VII would need to be further tested.

Among nine avirulent haplotypes, variable sites of three haplotypes (H6, H10, and H11) occurred in CDS and still kept the avirulent function. According to amino acid sequences of *AvrPiz-t* coding region, the 42nd amino acid of H6 was translated from GGU to GGC and encoded glycine (Gly), belonging to a synonymous mutation which doesn’t shift the avirulence function. The 2nd amino acid codon of H10 and H11 was mutated from glutamine (Gln) to stop codon and formed a nonsense mutation that led to the premature termination of peptide chain synthesis. But the 3rd codon of H10 and H11 is just right: a non-AUG start codon, UUG (Tikole and Sankararamakrishnan, 2006; Ivanov et al., 2011). So, we inferred that the translation of amino acid continued from the 3rd codon in H10 and H11, leading them to keep the avirulence function. Variable sites of other five novel *AvrPiz-t* avirulent haplotypes (H2, H4, H5, H8, and H9) occurred in non-CDS that didn’t affect the avirulent gene’s function.

**FIGURE 2:**
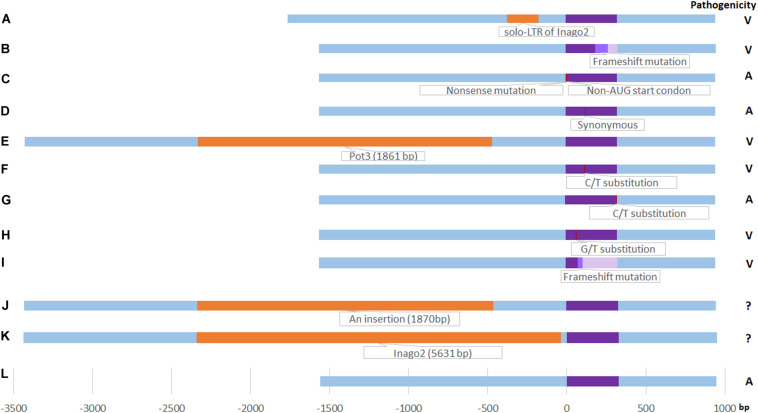
Schematic diagram of *AvrPiz-t* variants. **(A)** solo-LTR of Inago2 insertion in this study, orange color; (B): Frameshift mutation by an A insertion at 192 bp site of ORF in this study (light purple color), identical to Haplotype VII (Chen et al., 2014); **(C)**: Non-AUG-initiated N-terminal extension in this study (red color); **(D)** Synonymous mutation in this study (red color); **(E)**: Pot3 insertion (Li et al., 2009) (orange color); **(F)** C/T substitution at 122 bp site of ORF(A41V Substitution) (Li et al., 2009) (red color); **(G)** C/T substitution at 325 bp site of ORF in haplotype I (Chen et al., 2014) (red color); **(H)** G/T substitution at 68 bp site of ORF in haplotype II (Chen et al., 2014) (red color); **(I)** Frameshift mutation by a T del at 80 bp site of ORF in Haplotype VIII (Chen et al., 2014) (light purple color); **(J)** an 1870-bp insertion at 462 bp upstream of ORF (Chen et al., 2014) (orange color); **(K)** Inago2 insertion (5631 bp) at 41 bp upstream of ORF (Chen et al., 2014) (orange color), the length wasn’t drawn by scale for the frame limition; **(L)** EU837058 (Li et al., 2009) (purple color). Right column is the pathogenicity. V: Virulent; A: Avirulent. ?: Unknown.

In total, three novel variants (solo-LTR of retrotransposon Inago2 in promoter region, non-AUG-initiated N-terminal extension in CDS, and synonymous mutation) were found in *M. oryzae* field isolates of Yunnan in this study (Figure 2).

**FIGURE 3:**
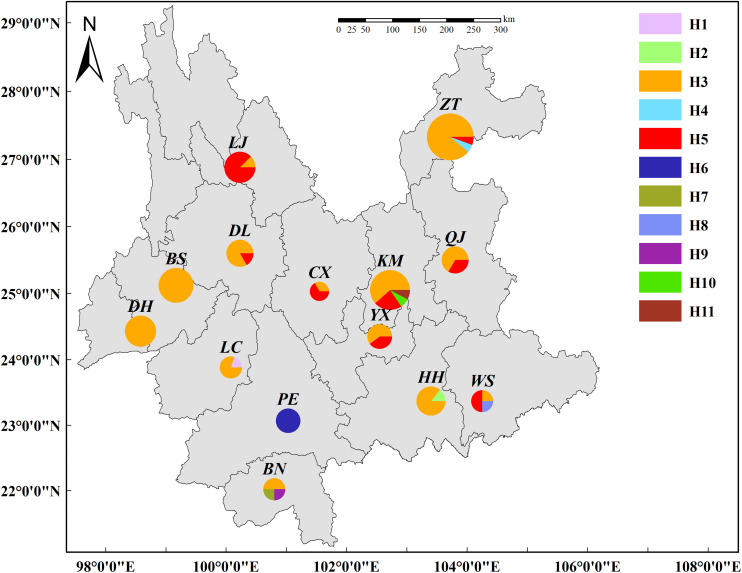
Distribution of *AvrPiz-t* haplotypes in *Magnaporthe oryzae* of Yunnan Province, China.

Of the 11 haplotypes, H3, H5, and H6 were composed of multiple isolates whereas residual haplotypes were only identified in a single isolate (Table 2). Haplotype distribution were mapped in the Yunnan Province of China (Figure 3). H3, identical with the original *AvrPiz-t* gene (EU837058), was comprised of 69% isolates and widely distributed in 13 prefectures, except for PE. H5 was comprised of 20% isolates in seven prefectures (KM, YX, CX, QJ, DL, LJ, and ZT), located in the north of Ailao Mountain. H6 with 3% isolates were only distributed in PE. Among the residential haplotypes, except for H7 and H9 in BN, H10 and H11 in KM, H1, H2, H4, H8 separately distributed in LC, HH, ZT, and WS.

### *AvrPiz-t* Haplotypes Diversity and Network

Genetic diversity at different regions/areas was calculated by using program DnaSP 5.10.01. Nucleotide diversity (π) among 14 prefectures ranged from 0 to 4.7 × 10^–4^, with an average of 2.0 × 10^–4^, and haplotype diversity (*H _*d*_*) ranged from 0 and 0.833, with an average of 0.487 (Table 3). WS holds the highest nucleotide diversity and haplotype diversity (π = 4.7 × 10^–4^, *H _*d*_* = 0.833) (Table 3). Among seven regions, the highest nucleotide diversity and haplotype diversity were in Southeastern Yunnan (π = 4.7 × 10^–4^, *H _*d*_* = 0.833), Southwestern followed. The nucleotide and haplotype diversity of isolates from *GJ* and *XI* rice growing area were similar. Haplotype numbers of the *XI* area was more than the *GJ* area. Genetic diversity is the basis of evolution and adaptation. Abundant genetic diversity makes populations more adaptable to the environment and more resistant to environmental changes and microbial invasion. Abundant diversity levels in the Southeastern and Southwestern Yunnan, *XI* area, indicated that resistant pressure in these areas might be higher. To adapt the resistant pressure, the *AvrPiz-t* gene of *M. oryzae* evolves diverse haplotypes to avoid *Piz-t* recognition and enable rice to survive.

**TABLE 3:** Frequency and diversity analysis of haplotype of Yunnan field *Magnaporthe oryzae* isolates in China.

**Location**	***No. samples***	**Haplotype frequency**											***No. haplotypes***	***H*_*d*_**	**π × *10*^−4^**
		**H1**	**H2**	**H3**	**H4**	**H5**	**H6**	**H7**	**H8**	**H9**	**H10**	**H11**			
Central	27	0	0	16	0	9	0	0	0	0	1	1	4	0.556	2.7
KM	13	0	0	8	0	3	0	0	0	0	1	1	4	0.603	3.3
YX	5	0	0	3	0	2	0	0	0	0	0	0	2	0.6	2.4
CX	3	0	0	1	0	2	0	0	0	0	0	0	2	0.667	2.7
QJ	6	0	0	4	0	2	0	0	0	0	0	0	2	0.533	2.1
Western	24	0	0	23	0	1	0	0	0	0	0	0	2	0.083	3
DL	6	0	0	5	0	1	0	0	0	0	0	0	2	0.333	1.3
BS	10	0	0	10	0	0	0	0	0	0	0	0	1	0	0
DH	8	0	0	8	0	0	0	0	0	0	0	0	1	0	0
Northwestern	8	0	0	1	0	7	0	0	0	0	0	0	2	0.25	1
LJ	8	0	0	1	0	7	0	0	0	0	0	0	2	0.25	1
Northeastern	18	0	0	16	1	1	0	0	0	0	0	0	3	0.216	0.8
ZT	18	0	0	16	1	1	0	0	0	0	0	0	3	0.216	0.8
South	11	0	1	8	0	0	0	1	0	1	0	0	4	0.491	1.5
HH	7	0	1	6	0	0	0	0	0	0	0	0	2	0.286	–
BN	4	0	0	2	0	0	0	1	0	1	0	0	3	0.833	4
Southwestern	8	1	0	4	0	0	3	0	0	0	0	0	3	0.679	2.1
LC	5	1	0	4	0	0	0	0	0	0	0	0	2	0.4	–
PE	3	0	0	0	0	0	3	0	0	0	0	0	1	0	0
Southeastern	4	0	0	1	0	2	0	0	1	0	0	0	3	0.833	4.7
WS	4	0	0	1	0	2	0	0	1	0	0	0	3	0.833	4.7
Total	100	1	1	69	1	20	3	1	1	1	1	1	11	0.487	2
*GJ*	69	0	0	48	1	18	0	0	0	0	1	1	5	0.446	2
*XI*	31	1	1	21	0	2	3	1	1	1	0	0	8	0.447	2
Total	100	1	1	69	1	20	3	1	1	1	1	1	11	0.487	2

*No samples: isolate numbers in haplotype analysis;No. haplotypes: numbers of haplotypes; H_*d*_: haplotype diversity; π: Nucleotide diversity; –: not analyzed.*

Overall, the neutrality test showed negative and insignificant values (*D* = −1.45377, *P* > 0.10; D^∗^ = −2.30204, *P* > 0.10) using Tajima’s D, Fu, and Li’s D^∗^(Table 4). Similar results were found in *GJ* and *XI* rice production areas; neutrality tests were all insignificant. These tests suggested that *AvrPiz-t* in Yunnan province conforms to the theory of neutral evolution.

**TABLE 4:** The neutrality test of Tajima’s D and Fu and Li’s D*tests based on *AvrPiz-t* of *Magnaporthe oryzae*.

**Region**	**Tajima’s D**	Fu and Li’s D*
Middle	–0.31256	–0.22854
West	–1.15933	–1.60583
Northwest	–1.05482	–1.12639
Northeast	–0.52899	0.66689
South	–1.42961	–1.65766
Southwest	1.1665	0.88779
Southeast	0.59158	0.59158
Total	–1.45377	–2.30204
*GJ*	–0.41172	–0.49716
*XI*	–1.62694	–1.50538

*Significant (*P* < 0.05).*

**FIGURE 4:**
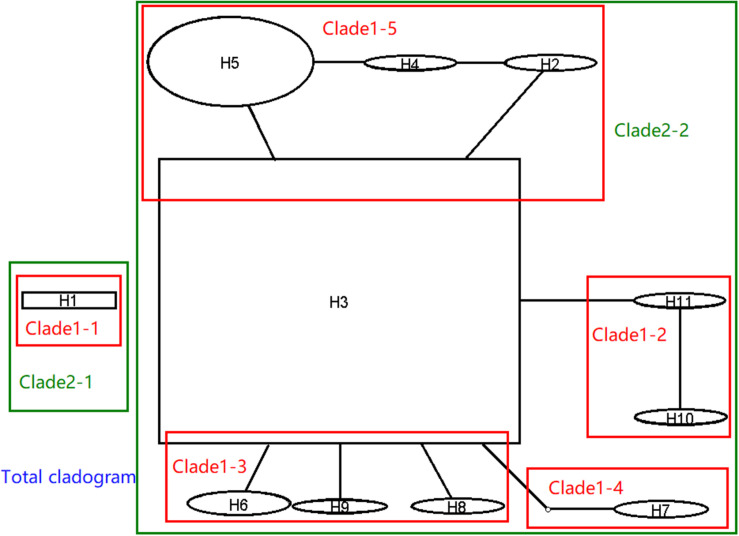
Network and nested cladogram of 11 *AvrPiz-t* haplotypes of *Magnaporthe oryzae* (black part: network; color part: nested cladogram).

In order to understand the evolutionary relationship of haplotypes of *M. oryzae*, the network of 11 haplotypes were analyzed. This was divided into the two main haplotype clades (Figure 4, black part). H1 separately formed a clade. The second clade was comprised of ten residential haplotypes. H3 was the central haplotype with high distributing frequency in the second clade, and the other nine haplotypes were originated directly or indirectly from H3. The network indicated that H1 and H3 were ancestral in the two clades. According to their sequences, however, H1 was originated from H3 through a retrotransposon insertion event at V5. H3 was inferred to be an ancestral haplotype.

Nested clade analysis was run to illuminate the possible history of *M. oryzae* populations based on the *AvrPiz-t* haplotype network. In the NCA, the network of 11 haplotypes of *AvrPiz-t* were nested by five clade 1-steps, two clade 2-steps, and total clade (Figure 4, color part). Clade 2–1 was separately comprised of clade1–1 only with H1. Clade 2–2 was comprised of clade 1–2, clade 1–3, clade 1–4, and clade 1–5. Nested clade analysis provided only limited information (Supplementary Tables S3, S4), but suggested a possible scenario of restricted dispersal by distance in the clade1–3 comprising haplotype H3, H6, H8, and H9 (H3 versus H6) and the clade 2–2 was comprised of haplotypes H2 to H11(H3 versus clade 1–3).

### Structure Variance of Virulent Isolates

In order to understand whether virulent isolates without *AvrPiz-t* PCR products (V-) are involved in structure variance, three pair primers were designed to amplify and sequence the three fragments (5′ fragment, middle fragment, and 3′ fragment including CDS region) of the *AvrPiz-t* gene of virulent isolates in this study. Among the total 39 V- isolates, 5′ fragment among 25 V- isolates and middle fragment among 23V- isolates were successfully amplified, but 3′ fragment among 39 V- isolates weren’t amplified. 18 5′ fragments and 16 middle fragments were sequenced and further assembled to EU837058. All 5′ fragments and five middle fragments were identical with the corresponding fragments of EU837058, and the other 11 fragments were sequenced disorderly. Then, the 11 fragments were cloned and sequenced. Blast comparison analysis was carried out in GenBank. It was found that nine sequences were homologous to a part sequence of cellobiose dehydrogenase of *M. oryzae* and two were homologous to a part sequence of *Enterobacter cloacae*, Therefore, the 11 fragments were inferred not to be target fragments. Checking the electrophoresis paragraphics, the nine fragments were slightly lower than the target fragments, and the two fragments were slightly bigger than the target fragments. Based on this, it can be judged whether the unsequenced fragment is the target fragment. Combining the amplification and sequence result, the 39 virulent isolates without *AvrPiz-t* products were defined to four genotypes (Table 5 and Supplementary Figure S4). There were 11 isolates with genotype “−−−” (28.2%), 16 with “+ −−” (41%), three with “− + −” (7.7%), and nine with “+ + −” (23.1%) out of the 39 virulent isolates. These results suggested that structure variance for absence (28.2%) or partial segment loss (71.8%) of *AvrPiz-t* gene in the field *M. oryzae* isolates may cause the avirulence function to be lost. In conclusion, 39 (95.1%) of the 41 virulent isolates in Yunnan were characterized by *AvrPiz-t* CDS deficiency. It indicated that the lack of *AvrPiz-t* gene CDS is the key factor leading to the loss of avirulent function.

**TABLE 5:** Genotypes and distribution of virulent isolates without *AvrPiz-t* gene in Yunnan Province.

**Genotype**	**No. virulent isolates (%)**	**KM**	**YX**	**QJ**	**CX**	**DL**	**BS**	**DH**	**LJ**	**ZT**	**HH**	**BN**	**LC**	**PE**	**WS**
“+ + +”	0(0)	0	0	0	0	0	0	0	0	0	0	0	0	0	0
“+ + −”	9(23.1)	2	0	0	0	0	0	0	0	1	0	0	5	1	0
“+ − +”	0(0)	0	0	0	0	0	0	0	0	0	0	0	0	0	0
“− + +”	0(0)	0	0	0	0	0	0	0	0	0	0	0	0	0	0
“+ −−”	16(41.0)	1	0	0	0	0	3	1	0	4	0	0	4	2	1
“− + −”	3(7.7)	1	0	0	0	0	0	1	0	0	1	0	0	0	0
“−− +”	0(0)	0	0	0	0	0	0	0	0	0	0	0	0	0	0
“−−−”	11(28.2)	0	0	0	0	0	1	7	0	0	1	2	0	0	0
Total	39	4	0	0	0	0	4	9	0	5	2	2	9	3	1

## Discussion

*Piz-t* is a valuable resistant gene to rice blast disease in China. It has been introduced to rice varieties to control blast. *Piz-t* gene was provided by donor parents Toride 1 and Toride 2 in rice blast resistant breeding. The two donor parents were developed from crosses of Indian varieties TKM1 and CO25, respectively, with Norin 8 (Yokoo, 2005). Utilizing Triode 1 as a cross parent, three excellent varieties for Yunnan Province of China were developed by the Japan and China joint breeding project in the 1990s (Yokoo, 2005). *Piz-t* held by Jinghua 9, which was one of major japonica rice varieties in Northeastern China, was formed from the hybrid combination of Toride 2 and Jingxi 17 by anther culture method (Ling et al., 1986). Eight japonica varieties (Jiang, 1995) and eight indigenous cultivars in the Yunnan Province of China (Zhu, 1988) also hold the *R* gene. 3.35% of varieties hold *Piz-t* gene among 328 indica hybrid rice varieties in the South of China (Wang W. J. et al., 2017). The corresponding *Avr* gene *AvrPiz-t* determines its stability and effectiveness under natural conditions. By pathogenicity assay, the knowledge of whether the isolate is pathogenic to *Piz-t* (avirulent or virulent) can be gained. 81.5% of 195 *M. oryzae* isolates were avirulent to *Piz-t* in Jiangxi Province (Lan et al., 2010), 85.2% of 467 isolates in Yunnan Province (Li et al., 2011), 34.4% of 87 isolate in Fujian Province (Yang et al., 2007), and 27.5% of 120 isolates in Hunan Province (Liu et al., 2016). By specific amplification of avirulent gene *AvrPiz-t*, the knowledge of whether the isolate holds the *AvrPiz-t* gene (+) or not (−) can be collected. The frequency of *AvrPiz-t* gene among 177 isolates was 55.9% in Heilongjiang Province (Li et al., 2012) and 84.6% among 26 isolates in Liaoning Province (Wang et al., 2014). Except for the limited value in Fujian and Hunan Province, *Piz-t* and *AvrPiz-t* are still a pair of valuable genes in Rice-*Magnaporthe* pathosystem in China. In our study, by *Avr* gene pathogenicity and amplification, the percentage of avirulent isolates with *AvrPiz-t* gene was shown to vary from 42.3% in the southwest to 83.3% in the northwest. The result suggests that these *M. oryza*e field isolates can be still beneficial to its correspondent resistant gene *Piz-t* in managing rice blast disease in Yunnan rice growing regions, except for the limited use in LC of Southwestern Yunnan.

According to Flor’s gene-for-gene theory, avirulent isolates with *AvrPiz-t* gene (A +) or virulent isolates without *AvrPiz-t* gene (V-) matches with the theory. 77.2% of isolates, including 64.7% A + isolates and 12.5% V- isolates, matched with gene-for-gene theory in a total of 312 isolates from 14 prefectures of seven regions in Yunnan Province (Table 1). These A + isolates triggered an immunity response (PTI and ETI) to induce IRBLzt-T (containing *Piz-t*) resistance to rice blast disease. And V- isolates did not trigger an immunity response in Rice-*Magnaporth*e pathosystem for the absence of *AvrPiz-t* gene. Additionally, 69 avirulent isolates without *AvrPiz-t* gene (A-) and two virulent isolates with *AvrPiz-t* gene (V +) were also screened by combining a pathogenicity assay and avirulent gene amplification. Because avirulent frequency was evaluated by either pathogenicity assay or *Avr* gene amplification methods, there was some deviation, so the combining method better improved the precision of frequency of avirulent isolates with the *AvrPiz-t* gene. Similar methods had been applied for the analysis of *AvrPita1* (Dai et al., 2010), *AvrPib* (Zhang et al., 2015), and *AvrPii* (Lu et al., 2019). 69 isolates were not pathogenic to IRBLzt-T and didn’t hold the *AvrPiz-t* gene. It indicated these isolates did not match the *AvrPiz-t* gene for *Piz-t* gene fashion. Maybe other *Avr*/*R* gene pairs played a role in the resistance to these 69 isolates. Another explanation was that the primer binding sites among these 69 A- isolates were possibly significantly altered and resulted in the failure of the gene amplification. 2 V + isolates in this study suggested that the function of their *AvrPiz-t* gene alleles had been lost. Sequences of V + isolates were further analyzed to clarify the relationship of their variation and virulence.

During its long coevolutionary history, the *Piz-t* gene from rice and *AvrPiz-t* gene from *M. oryzae* have experienced a mutual arm race to avoid recognition, resulting in diverse *Piz-t* and *AvrPiz-t* genes. A total number of 565 polymorphic sites and 46 haplotypes were identified in the 2.9 kb sequence of all the *Piz-t* alleles in forty-five rice land races in Indian (Thakur et al., 2013). Seventeen *Piz-t* allelic types were identified among 24 cultivars in China (Wang Y. et al., 2016). Higher variance levels of *Piz-t* gene suggested that *AvrPiz-t* gene experienced some degree of selection pressure. Four haplotypes (I, II, VII, and VIII) in rice isolates, three haplotypes (III, IV, and V) in non-rice isolates, and one haplotype (VI) shared by distant isolates in the phylogeny were identified in ORFs (327 bp) among 711 isolates of *M. oryzae* collected from 38 countries and regions (Chen et al., 2014). Four TE insertions on promoter regions were identified in these isolates. A strong positive selection is detected at the locus. *M. oryzae* evades recognition of *Piz-t* gene in rice through diversification of its coding sequence and TEs in the promoter region of *AvrPiz-t* (Chen et al., 2014). In this study, at least nine haplotypes were identified based on *AvrPiz-t* gene (non-CDS and CDS, 2507 bp) from 312 *M. oryzae* isolates in Yunnan. However, the neutrality tests suggested that *AvrPiz-t* in Yunnan province conforms to the theory of neutral evolution. The result indicated the evolutionary history of *AvrPiz-t* and *Piz-t* in Yunnan was different from the previous study (Chen et al., 2014). The genetic diversity and variance of *Piz-t* gene in Yunnan has not yet been reported. And whether *AvrPiz-t* faced smaller resistance pressure from *Piz-t* in Yunnan would need to be tested through further study.

In driving pathogenicity variation and genomic plasticity, point mutation, deletion, frameshift, and transposable elements (TEs) of *Avr* genes play a key role among a lot of plant pathogenic fungi (Joosten et al., 1994; Schurch et al., 2004; Dodds et al., 2006; Ridout et al., 2006; Raffaele and Kamoun, 2012; Faino et al., 2016). Analysis of *Avr* gene DNA sequences can help to understand its variable pattern, pathogenicity mechanism, and evolution. It was reported that a Pot3 insertion or single nucleotide substitution resulting in virulence shift of *AvrPiz-t* (Li et al., 2009). Four haplotypes (I, II, VII, and VIII) in rice isolates were identified in AvrPiz-t ORF (Chen et al., 2014). At least two kinds of insertion exists in Chinese isolates, such as Inago2 insertion at 41 bp upstream ORF and a 1870 bp insertion at 462 bp of upstream ORF (Chen et al., 2014). Except for the VII haplotype, just as with ORF of H7 in the study, the other variants were not found in this study (Figure 2). Here, we found one novel virulent variant in the *AvrPiz-t* gene and two novel avirulent variants in *M. oryzae* field isolates of Yunnan (Figure 2). The insertion of solo-LTR of Inago2 in the promoter region of *AvrPiz-t* was founded in virulent isolate 07-231-2a in this study (Figure 2A). Genetic changes led by transposons in *M. oryzae* have been predicted to be responsible for pathogenic variation (Valent and Chumley, 1991; Kang et al., 2001; Zhou et al., 2007). The insertion of a 1.9 kb MINE retrotransposon in the last exon of *ACE1* was hypothesized to be responsible for the shift from avirulence to virulence (Fudal et al., 2005). Transposon in the genomic regions near *AVR-Pia* and *AVR-Pii* increased the probability of gene loss and horizontal transfer (Silva et al., 2004; Rehmeyer et al., 2006). An insertion of Mg-SINE in *AvrPi9* resulting in the loss of avirulence function was identified in the derivative virulent strain (Wu et al., 2015). A Pot3 transposon in the encoding region of *Avr-Pita* was identified from a field virulent isolate (Zhou et al., 2007). TE insertion in 108 *M. oryzae* isolates were identified to lead to loss of the avirulence function of *AvrPib* (Zhang et al., 2015). These studies showed that transposons are responsible for the instability of *Avr* genes in *M. oryzae* and is one pathogenic mechanism to defeat rice corresponding *R* genes. Solo-LTR of retrotransposon Inago2 provides another example for the association of retrotransposon elements with *Avr* genes in this study. Frameshift mutations are caused by indels of a base (or bases) in the CDS region, that result in a distinguished different translation of the message beside the site of the mutation (Streisinger et al., 1966). It is predicted to import an aberrant produce and a truncated protein, and alter gene function. In a virulent isolate of *M. oryzae*, a frameshift mutation caused by two-base-pair insertions of *AvrPi-ta1* has been identified (Dai et al., 2010). In this study, an adenine insertion in *AvrPiz-t* CDS region caused a frameshift mutation in the virulent isolate 08-39-2d (Figure 2B). The frameshift mutation is similar to VII (Chen et al., 2014) and leads the isolate 08-39-2d to be virulent to *Piz-t*. 07-231-2a holding the insertion of solo-LTR of Inago2 in the promoter region of *AvrPiz-t* and 08-39-2d holding the frameshift mutation in *AvrPiz-t* CDS region were virulent to Piz-t by pathogenicity assay, however, whether the two mutations would affect gene expression needs to be further validated by reconstruction of mutants and transcript level.

Structure variances of the *Avr* gene, such as absent or partial deletion, may lead the gene’s function to be shifted. Analysis of the structure variance of all 39 virulent isolates without *AvrPiz-t* by fragment amplification in this study showed that absence or partial segment loss may result in the function of *AvrPiz-t* gene being lost. Similar results were found in other *Avr* genes of *M. oryzae*. Absence of *AVR1-CO39* in isolate Guy11 caused the avirulent function for *Pi-CO39* to be lost (Farman and Leong, 1998). At the *AVR1-CO39* locus in *M. oryzae*, three types (G1, G2, and J) of diversification were reported (Farman et al., 2002). An approximate deletion of 20 kb was found in the G types, as well as a deletion of the 5′ half of *AVR1-CO39* to point mutations in J type. *AVR-Pia* and *AVR-Pii* showed gain/loss mutations (Yoshida et al., 2009). *Avr-Pik/km/kp* showed presence/absence polymorphisms (Yoshida et al., 2009). Segmental deletion and complete absence were identified to lead to loss of the avirulence function of *AvrPib* (Zhang et al., 2015). So, absence or partial deletion of the *Avr* gene is also a major pathogenesis of *M. oryzae*.

In eukaryotes, translation initiation generally starts at the AUG codon nearest to the mRNA 5′ cap. However, in some cases, initiation can start at codons distinguished from AUG by a single nucleotide, especially the near-cognate codons AUA, AUU, ACG, CUG, UUG, and GUG (Ivanov et al., 2011). In mammals, 38 mRNAs from 23 genes have non-AUG initiation codons in GenBank (Tikole and Sankararamakrishnan, 2006). N-terminal extensions initiated by novel conserved potential non-AUG start codon were identified in 42 human genes (Ivanov et al., 2011). At least 47 non-canonical start codons existed in *Escherichia coli*, each of which could initiate the protein synthesis (Hecht et al., 2017). In this study, although the 2nd amino acid of H10 and H11 is mutated into the stop codon, the 3rd codon UUG, a near-cognate codon, were inferred to act as a start codon and initiated N-terminal extensions. The case of non-AUG-initiated N-terminal extension is first reported in *M. oryzae* field isolates. Maybe it is a new mechanism for *Avr* gene to keep avirulence function. Signal peptide missing 2 amino acids in H10 and H11 would be further investigated to see whether they affect AvrPiz-t secretion.

According to the network of *AvrPiz-t* haplotypes in this study, H3 was an ancestral haplotype found in Yunnan Province of China. Combining the distribution of haplotypes, it was found that H5, which was originated from H3, was only distributed in the north of the Ailao Mountain. Ailao Mountain is located on a low-latitude plateau in southwestern China. It runs through central Yunnan from Northwest to Southeast and is located at the junction of the three natural geographical regions of the Yunnan-Guizhou Plateau, the Hengduan Mountain, and the Qinghai-Tibetan Plateau in China. It is the geographical boundary between the Western Yunnan Rift Valley and the central Yunnan Plateau. It is also the winter climate boundary line between warm western and cold eastern Yunnan (Wu and Wang, 1983; Comprehensive Survey Group of the Ailao Mountains Natural Reserve, 1988; Wang, 2002; Zhu and Yan, 2009). Its particular geographical location makes the Ailao Mountain an important traffic thoroughfare for animals and plants. The Ailao Mountain plays an important role in species distribution pattern. Although NCA didn’t provide useful information on population history, based on the fact that H5 was only distributed in the north of the Ailao Mountain, it was assumed that the Ailao Mountain may have an effect on the distribution pattern of *M. oryzae AvrPiz-t* haplotypes in Yunnan Province.

## Conclusion

Sophisticated strategies have been developed for plant pathogens and their hosts to escape each other’s defense systems in their coevolution. In summary, the high frequency of *M. oryza*e avirulent isolates with *AvrPiz-t* gene and abundant avirulent haplotypes both suggest that *Piz-t* and *AvrPiz-t* are a useful pair of *R-Avr* genes in Rice-*Magnaporthe* pathosystem of Yunnan Province of China. Avoiding rice *R* gene recognition to result in rice disease, some new virulent isolates of *M. oryzae* have emerged to defeat the corresponding *Piz-t* gene by the solo-LTR insertion of retrotransposon inago2 in promoter region, the frameshift mutation, and structure variance of *AvrPiz-t* in the field of Yunnan, China. These novel virulent variants and structure variance of *AvrPiz-t* act as a major evolutionary force. And abundant *AvrPiz-t* haplotypes a play powerful stabilizing role for *Piz-t* as a broad spectrum resistance gene. These results provide valuable information for rice blast control in the Rice-*Magnaporthe* pathosystem and knowledge on *Avr* gene evolution mechanism.

## Data Availability Statement

The raw data supporting the conclusions of this article will be made available by the authors, without undue reservation, to any qualified researcher.

## Author Contributions

JL designed the research and revised the manuscript. QW, JL, LL, and CH performed the experiments. QW, JL, and CL analyzed the data. QW wrote the manuscript. All authors contributed to the article and approved the submitted version.

## Conflict of Interest

The authors declare that the research was conducted in the absence of any commercial or financial relationships that could be construed as a potential conflict of interest.

## Footnotes

1 http://darwin.uvigo.es/
