# Supplementary material for: Novel Variation and Evolution of AvrPiz-t of Magnaporthe oryzae in Field Isolates
Source: Front Genet. 2020 Aug 28;11:746. doi: 10.3389/fgene.2020.00746 (PMC7484972; doi:10.3389/fgene.2020.00746)
Supplement: Supplementary file 1 [file Data_Sheet_1.docx]

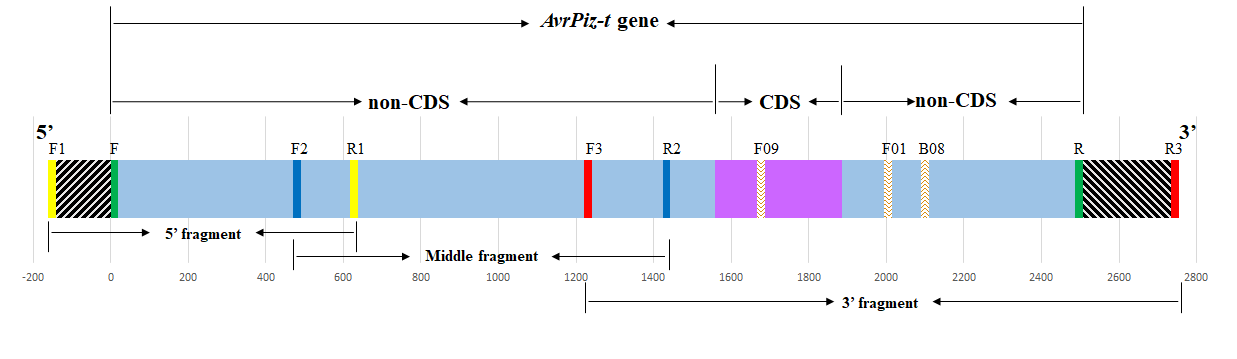


**Figure S1.** Location of Primers used in this study, for amplification and sequence of full gene and fragments of *AvrPiz-t*

F: AvztF R: AvztR F1: AvrPizt-F1 R1: AvrPizt-R1 F2: AvrPizt-F2 R2: AvrPizt-R2 F3: AvrPizt-F3 R3: AvrPizt-R3 F01: C8-W1F-F01 F09: F3-W1F-F09 B08: C8-CW1F-B08


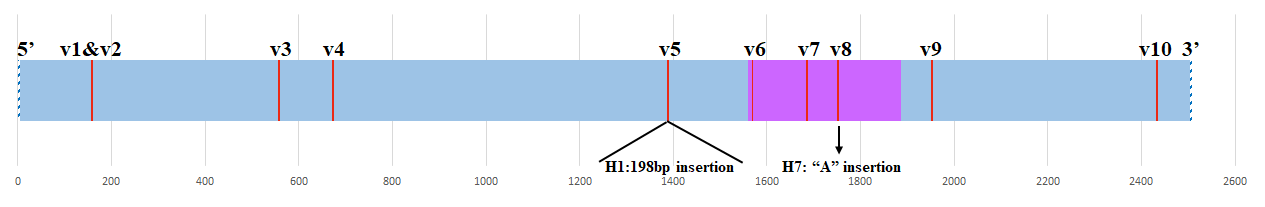


**Figure S2.** Location of variable sites in the *AvrPiz-t* gene (EU837058)

Purple is CDS, light blue is non-coding regions


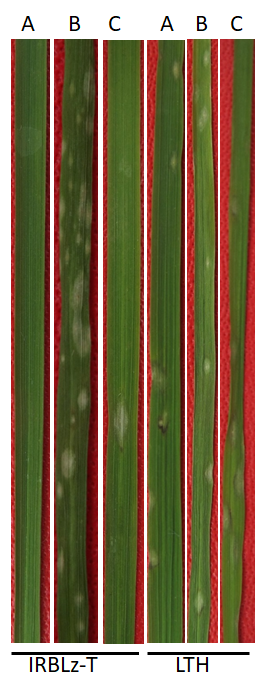


**Figure S3**. Disease reaction of rice cultivars to *Magnaporthe oryzae* isolates.

A: 11-64-1a B: 07-231-2a C: 08-39-2d

**
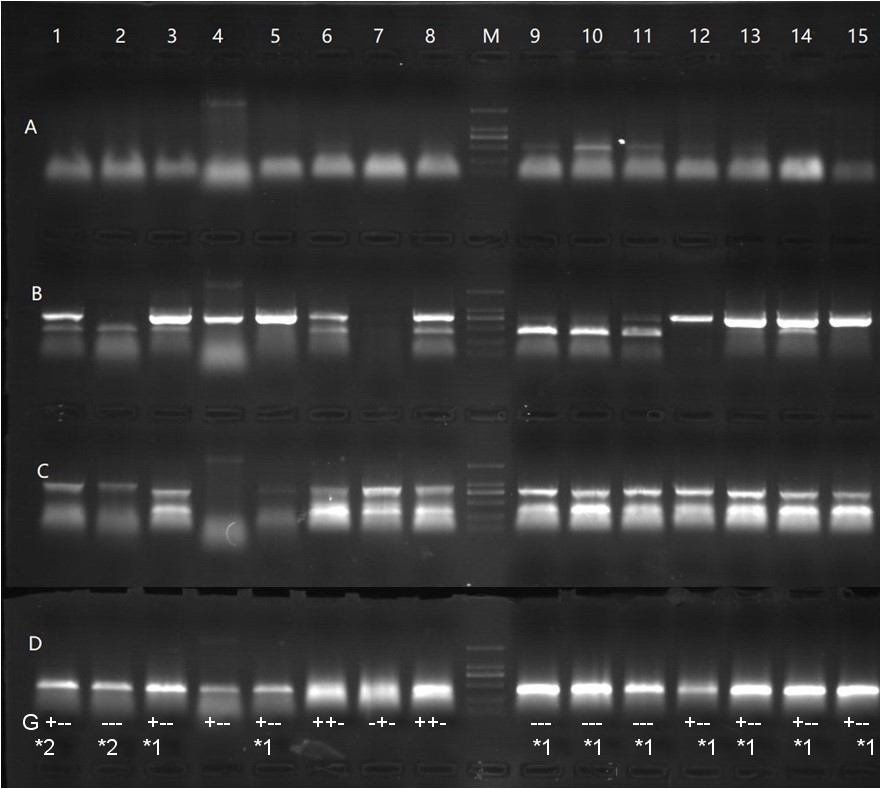
**

**Figure S4.** Structure variance for *AvrPiz-t* gene of partial *Magnaporthe oryzae* virulent isolates without *AvrPiz-t* gene

A: *AvrPiz-t* gene amplificon B: 5’fragment amplicon of *AvrPiz-t* C: Middle fragment amplicon of *AvrPiz-t* D：3’ fragment amplicon of *AvrPiz-t* G: genotype of three fragment amplicons (5’, middle and 3’ fragments) of AvrPiz-t gene among some *Magnaporthe oryzae* virulent isolates without *AvrPiz-t* gene 1 to 15 is respectively given to isolate 95-52-1b，95-53-1a，07-230-1a，07-325-1a，07-231-1a，07-14-1a，07-14-4a，94-70-1c，95-52-1c，95-53-1c，95-53-1e，95-71-2a，95-71-2b，96-20-2a，and 97-47-1. M：Size marker, representative fragment 2000bp, 1000bp, 750bp, 500bp, 250bp, 100bp. *1: non target middle fragment, homologous to part sequence of cellobiose dehydrogenase of *M.oryzae* *2: non target middle fragment, homologous to part sequence of *Enterobacter cloacae.*
