# Supplementary material for: Novel Variation and Evolution of AvrPiz-t of Magnaporthe oryzae in Field Isolates
Source: Front Genet. 2020 Aug 28;11:746. doi: 10.3389/fgene.2020.00746 (PMC7484972; doi:10.3389/fgene.2020.00746)
Supplement: Supplementary file 2 [file Table_1.docx]

**Table S1.** Isolates information, Pathogenicity, *AvrPiz-t* amplification and genotype of virulent isolate

| **Isolate (Year)** | **Prefecture(abbr.)** | **Production area^a^** | **IRBLzt-T（Pizt）** | ***AvrPiz-t*** | **Haplotype** | **Genotype of virulent isolate** |
| --- | --- | --- | --- | --- | --- | --- |
| Central |  |  |  |  |  |  |
| 07-14-3a (2007) | Kunming(KM) | *GJ* | A | + |  |  |
| 07-15-1a (2007) | Kunming(KM) | *GJ* | A | + | H11 |  |
| 07-15-3a (2007) | Kunming(KM) | *GJ* | A | + | H10 |  |
| 07-8-1a (2007) | Kunming(KM) | *GJ* | A | + | H03 |  |
| 93-1-2a (1993) | Kunming(KM) | *GJ* | A | + |  |  |
| 93-9-1a (1993) | Kunming(KM) | *GJ* | A | + | H03 |  |
| 94-120-1a (1994) | Kunming(KM) | *GJ* | A | + | H05 |  |
| 94-121-2a (1994) | Kunming(KM) | *GJ* | A | + | H05 |  |
| 94-121-2b (1994) | Kunming(KM) | *GJ* | A | + |  |  |
| 96-1-1a (1996) | Kunming(KM) | *GJ* | A | + | H03 |  |
| 96-1-1b (1996) | Kunming(KM) | *GJ* | A | + |  |  |
| 96-2-1a (1996) | Kunming(KM) | *GJ* | A | + | H03 |  |
| 96-2-1b (1996) | Kunming(KM) | *GJ* | A | + |  |  |
| 96-2-2b (1996) | Kunming(KM) | *GJ* | A | + | H03 |  |
| 97-105-1a (1997) | Kunming(KM) | *GJ* | A | + | H05 |  |
| 97-106-1a (1997) | Kunming(KM) | *GJ* | A | + | H03 |  |
| 97-107-1a (1997) | Kunming(KM) | *GJ* | A | + | H03 |  |
| 97-108-1a (1997) | Kunming(KM) | *GJ* | A | + | H03 |  |
| 07-14-1a (2007) | Kunming(KM) | *GJ* | V | - |  | "+ + -" |
| 07-14-2a (2007) | Kunming(KM) | *GJ* | V | - |  | "+ + -" |
| 07-14-4a (2007) | Kunming(KM) | *GJ* | V | - |  | "- + -" |
| 93-7-1a (1993) | Kunming(KM) | *GJ* | V | - |  | "+ - -" |
| 02-18-1e (2002) | Kunming(KM) | *GJ* | A | - |  |  |
| 07-1-1a (2007) | Kunming(KM) | *GJ* | A | - |  |  |
| 97-105-1b (1997) | Kunming(KM) | *GJ* | A | - |  |  |
| 97-106-2c (1997) | Kunming(KM) | *GJ* | A | - |  |  |
| YJW-1-1C④ (2012) | Yuxi(YX) | *GJ* | A | - |  |  |
| YJW-1-1b (2012) | Yuxi(YX) | *GJ* | A | + | H03 |  |
| YJW-2-1d (2012) | Yuxi(YX) | *GJ* | A | + | H03 |  |
| 93-2-2a (1993) | Yuxi(YX) | *GJ* | A | + | H05 |  |
| 93-4-1a (1993) | Yuxi(YX) | *GJ* | A | + | H05 |  |
| 93-4-1b (1993) | Yuxi(YX) | *GJ* | A | + |  |  |
| 93-4-2a (1993) | Yuxi(YX) | *GJ* | A | + |  |  |
| 93-4-2c (1993) | Yuxi(YX) | *GJ* | A | + | H03 |  |
| 07-160-1a (2007) | Qujing(QJ) | *GJ* | A | + | H05 |  |
| 07-160-2b (2007) | Qujing(QJ) | *GJ* | A | + |  |  |
| 07-161-1a (2007) | Qujing(QJ) | *GJ* | A | + | H05 |  |
| 96-4-1a (1996) | Qujing(QJ) | *GJ* | A | + | H03 |  |
| 97-100-1a (1997) | Qujing(QJ) | *GJ* | A | + |  |  |
| 97-100-2a (1997) | Qujing(QJ) | *GJ* | A | + | H03 |  |
| 97-101-1a (1997) | Qujing(QJ) | *GJ* | A | + |  |  |
| 97-101-2a (1997) | Qujing(QJ) | *GJ* | A | + |  |  |
| 97-102-1a (1997) | Qujing(QJ) | *GJ* | A | + |  |  |
| 97-102-1b (1997) | Qujing(QJ) | *GJ* | A | + |  |  |
| 97-97-1a (1997) | Qujing(QJ) | *GJ* | A | + | H03 |  |
| 97-98-1a (1997) | Qujing(QJ) | *GJ* | A | + | H03 |  |
| 11-64-1a (2011) | Chuxiong(CX) | *GJ* | A | + | H05 |  |
| 11-82-1b (2011) | Chuxiong(CX) | *GJ* | A | + | H05 |  |
| J32-7 (2010) | Chuxiong(CX) | *GJ* | A | + | H03 |  |
| Western |  |  |  |  |  |  |
| 08-05-3a (2008) | Dali(DL) | *GJ* | A | + | H03 |  |
| 08-06-3a (2008) | Dali(DL) | *GJ* | A | + | H05 |  |
| 08-09-1a (2008) | Dali(DL) | *GJ* | A | + | H03 |  |
| 08-16-1a (2008) | Dali(DL) | *GJ* | A | + | H03 |  |
| 08-17-1a (2008) | Dali(DL) | *GJ* | A | + | H03 |  |
| 08-22-1a (2008) | Dali(DL) | *GJ* | A | + | H03 |  |
| 94-200-1a (1994) | Baoshan(BS) | *GJ* | A | + | H03 |  |
| 95-10-3a (1995) | Baoshan(BS) | *GJ* | A | + | H03 |  |
| 95-11-1a (1995) | Baoshan(BS) | *GJ* | A | + | H03 |  |
| 95-12-1a (1995) | Baoshan(BS) | *GJ* | A | + | H03 |  |
| 95-12-1b (1995) | Baoshan(BS) | *GJ* | A | + |  |  |
| 95-12-1d (1995) | Baoshan(BS) | *GJ* | A | + |  |  |
| 95-12-2c (1995) | Baoshan(BS) | *GJ* | A | + |  |  |
| 95-12-3a (1995) | Baoshan(BS) | *GJ* | A | + |  |  |
| 95-12-3b (1995) | Baoshan(BS) | *GJ* | A | + |  |  |
| 95-13-1b (1995) | Baoshan(BS) | *GJ* | A | + |  |  |
| 95-14-1a (1995) | Baoshan(BS) | *GJ* | A | + |  |  |
| 95-14-1b (1995) | Baoshan(BS) | *GJ* | A | + |  |  |
| 95-14-1c (1995) | Baoshan(BS) | *GJ* | A | + |  |  |
| 95-15-1b (1995) | Baoshan(BS) | *GJ* | A | + |  |  |
| 95-15-1d (1995) | Baoshan(BS) | *GJ* | A | + |  |  |
| 95-15-1e (1995) | Baoshan(BS) | *GJ* | A | + |  |  |
| 95-16-1b (1995) | Baoshan(BS) | *GJ* | A | + |  |  |
| 95-16-1d (1995) | Baoshan(BS) | *GJ* | A | + |  |  |
| 95-16-2a (1995) | Baoshan(BS) | *GJ* | A | + |  |  |
| 95-16-2b (1995) | Baoshan(BS) | *GJ* | A | + |  |  |
| 95-16-3a (1995) | Baoshan(BS) | *GJ* | A | + |  |  |
| 95-18-1a (1995) | Baoshan(BS) | *GJ* | A | + |  |  |
| 95-19-1a (1995) | Baoshan(BS) | *GJ* | A | + |  |  |
| 95-21-1a (1995) | Baoshan(BS) | *GJ* | A | + | H03 |  |
| 95-2-1a (1995) | Baoshan(BS) | *GJ* | A | + | H03 |  |
| 95-2-2a (1995) | Baoshan(BS) | *GJ* | A | + |  |  |
| 95-23-4a (1995) | Baoshan(BS) | *GJ* | A | + |  |  |
| 95-23-4c (1995) | Baoshan(BS) | *GJ* | A | + |  |  |
| 95-23-4d (1995) | Baoshan(BS) | *GJ* | A | + | H03 |  |
| 95-23-5a (1995) | Baoshan(BS) | *GJ* | A | + |  |  |
| 95-31-2a (1995) | Baoshan(BS) | *GJ* | A | + |  |  |
| 95-54-1a (1995) | Baoshan(BS) | *GJ* | A | + |  |  |
| 95-54-2a (1995) | Baoshan(BS) | *GJ* | A | + |  |  |
| 95-55-1a (1995) | Baoshan(BS) | *GJ* | A | + |  |  |
| 95-56-1a (1995) | Baoshan(BS) | *GJ* | A | + |  |  |
| 95-7-2c (1995) | Baoshan(BS) | *GJ* | A | + |  |  |
| 95-8-1a (1995) | Baoshan(BS) | *GJ* | A | + |  |  |
| 95-8-1b (1995) | Baoshan(BS) | *GJ* | A | + |  |  |
| 95-8-1e (1995) | Baoshan(BS) | *GJ* | A | + |  |  |
| 95-8-2b (1995) | Baoshan(BS) | *GJ* | A | + |  |  |
| 95-8-3a (1995) | Baoshan(BS) | *GJ* | A | + |  |  |
| 95-8-3d (1995) | Baoshan(BS) | *GJ* | A | + |  |  |
| 95-8-5d (1995) | Baoshan(BS) | *GJ* | A | + |  |  |
| 95-8-5e (1995) | Baoshan(BS) | *GJ* | A | + |  |  |
| 95-9-1a (1995) | Baoshan(BS) | *GJ* | A | + |  |  |
| 95-9-3a (1995) | Baoshan(BS) | *GJ* | A | + | H03 |  |
| 96-14-1b (1996) | Baoshan(BS) | *GJ* | A | + |  |  |
| 96-16-2a (1996) | Baoshan(BS) | *GJ* | A | + |  |  |
| 96-17-1a (1996) | Baoshan(BS) | *GJ* | A | + |  |  |
| 96-21-1a (1996) | Baoshan(BS) | *GJ* | A | + | H03 |  |
| 96-8-1a (1996) | Baoshan(BS) | *GJ* | A | + |  |  |
| 96-9-1a (1996) | Baoshan(BS) | *GJ* | A | + | H03 |  |
| 95-13-1A (1995) | Baoshan(BS) | *GJ* | V | - |  | "+ - -" |
| 96-11-1a (1996) | Baoshan(BS) | *GJ* | V | - |  | "- - -" |
| 96-20-1b (1996) | Baoshan(BS) | *GJ* | V | - |  | "+ - -" |
| 96-20-2a (1996) | Baoshan(BS) | *GJ* | V | - |  | "+ - -"^*1^ |
| 08-43-1a (2008) | Baoshan(BS) | *GJ* | A | - |  |  |
| 94-201-1a (1994) | Baoshan(BS) | *GJ* | A | - |  |  |
| 95-10-5b (1995) | Baoshan(BS) | *GJ* | A | - |  |  |
| 95-10-6b (1995) | Baoshan(BS) | *GJ* | A | - |  |  |
| 95-10-6c (1995) | Baoshan(BS) | *GJ* | A | - |  |  |
| 95-10-7a (1995) | Baoshan(BS) | *GJ* | A | - |  |  |
| 95-10-7b (1995) | Baoshan(BS) | *GJ* | A | - |  |  |
| 95-12-1c (1995) | Baoshan(BS) | *GJ* | A | - |  |  |
| 95-12-2a (1995) | Baoshan(BS) | *GJ* | A | - |  |  |
| 95-12-2b (1995) | Baoshan(BS) | *GJ* | A | - |  |  |
| 95-15-1c (1995) | Baoshan(BS) | *GJ* | A | - |  |  |
| 95-15-2a (1995) | Baoshan(BS) | *GJ* | A | - |  |  |
| 95-16-1a (1995) | Baoshan(BS) | *GJ* | A | - |  |  |
| 95-18-3b (1995) | Baoshan(BS) | *GJ* | A | - |  |  |
| 95-18-3c (1995) | Baoshan(BS) | *GJ* | A | - |  |  |
| 95-22-1a (1995) | Baoshan(BS) | *GJ* | A | - |  |  |
| 95-22-3a (1995) | Baoshan(BS) | *GJ* | A | - |  |  |
| 95-23-2b (1995) | Baoshan(BS) | *GJ* | A | - |  |  |
| 95-23-3b (1995) | Baoshan(BS) | *GJ* | A | - |  |  |
| 95-7-1b (1995) | Baoshan(BS) | *GJ* | A | - |  |  |
| 95-8-1d (1995) | Baoshan(BS) | *GJ* | A | - |  |  |
| 95-8-3b (1995) | Baoshan(BS) | *GJ* | A | - |  |  |
| 95-8-3c (1995) | Baoshan(BS) | *GJ* | A | - |  |  |
| 95-9-2b (1995) | Baoshan(BS) | *GJ* | A | - |  |  |
| 95-9-2c (1995) | Baoshan(BS) | *GJ* | A | - |  |  |
| 95-9-4c (1995) | Baoshan(BS) | *GJ* | A | - |  |  |
| 96-10-1a (1996) | Baoshan(BS) | *XI* | A | - |  |  |
| 95-24-1a (1995) | Dehong(DH) | *XI* | A | + |  |  |
| 95-24-1b (1995) | Dehong(DH) | *XI* | A | + |  |  |
| 95-25-1a (1995) | Dehong(DH) | *XI* | A | + | H03 |  |
| 95-26-2b (1995) | Dehong(DH) | *XI* | A | + |  |  |
| 95-33-1b (1995) | Dehong(DH) | *XI* | A | + | H03 |  |
| 95-33-2a (1995) | Dehong(DH) | *XI* | A | + |  |  |
| 95-34-1a (1995) | Dehong(DH) | *XI* | A | + | H03 |  |
| 95-34-1b (1995) | Dehong(DH) | *XI* | A | + |  |  |
| 95-34-2b (1995) | Dehong(DH) | *XI* | A | + | H03 |  |
| 95-35-1b (1995) | Dehong(DH) | *XI* | A | + |  |  |
| 95-36-1a (1995) | Dehong(DH) | *XI* | A | + | H03 |  |
| 95-38-1a (1995) | Dehong(DH) | *XI* | A | + |  |  |
| 95-38-1b (1995) | Dehong(DH) | *XI* | A | + |  |  |
| 95-42-3a (1995) | Dehong(DH) | *XI* | A | + | H03 |  |
| 95-42-3b (1995) | Dehong(DH) | *XI* | A | + |  |  |
| 95-43-1b (1995) | Dehong(DH) | *XI* | A | + |  |  |
| 95-45-1b (1995) | Dehong(DH) | *XI* | A | + | H03 |  |
| 95-45-2a (1995) | Dehong(DH) | *XI* | A | + |  |  |
| 95-46-1a (1995) | Dehong(DH) | *XI* | A | + |  |  |
| 95-46-2a (1995) | Dehong(DH) | *XI* | A | + |  |  |
| 95-46-3b (1995) | Dehong(DH) | *XI* | A | + |  |  |
| 95-48-1a (1995) | Dehong(DH) | *XI* | A | + |  |  |
| YN153 (1995) | Dehong(DH) | *XI* | A | + | H03 |  |
| 95-42-2a (1995) | Dehong(DH) | *XI* | V | - |  | "- - -" |
| 95-45-2b (1995) | Dehong(DH) | *XI* | V | - |  | "- + -" |
| 95-46-1b (1995) | Dehong(DH) | *XI* | V | - |  | "- - -" |
| 95-52-1b (1995) | Dehong(DH) | *XI* | V | - |  | "+ - -"^*2^ |
| 95-52-1c (1995) | Dehong(DH) | *XI* | V | - |  | "- - -"^*1^ |
| 95-53-1a (1995) | Dehong(DH) | *XI* | V | - |  | "- - -"^*2^ |
| 95-53-1b (1995) | Dehong(DH) | *XI* | V | - |  | "- - -" |
| 95-53-1c (1995) | Dehong(DH) | *XI* | V | - |  | "- - -"^*1^ |
| 95-53-1e (1995) | Dehong(DH) | *XI* | V | - |  | "- - -"^*1^ |
| 95-25-2a (1995) | Dehong(DH) | *XI* | A | - |  |  |
| 95-25-5a (1995) | Dehong(DH) | *XI* | A | - |  |  |
| 95-26-1b (1995) | Dehong(DH) | *XI* | A | - |  |  |
| 95-26-1c (1995) | Dehong(DH) | *XI* | A | - |  |  |
| 95-33-1a (1995) | Dehong(DH) | *XI* | A | - |  |  |
| 95-33-2d (1995) | Dehong(DH) | *XI* | A | - |  |  |
| 95-33-3a (1995) | Dehong(DH) | *XI* | A | - |  |  |
| 95-33-3b (1995) | Dehong(DH) | *XI* | A | - |  |  |
| 95-42-1b (1995) | Dehong(DH) | *XI* | A | - |  |  |
| 95-43-2b (1995) | Dehong(DH) | *XI* | A | - |  |  |
| 95-46-1c (1995) | Dehong(DH) | *XI* | A | - |  |  |
| 95-48-1b (1995) | Dehong(DH) | *XI* | A | - |  |  |
| 95-48-1d (1995) | Dehong(DH) | *XI* | A | - |  |  |
| 95-48-1e (1995) | Dehong(DH) | *XI* | A | - |  |  |
| 95-52-2b (1995) | Dehong(DH) | *XI* | A | - |  |  |
| D-1-1g (2010) | Dehong(DH) | *XI* | A | - |  |  |
| Northwestern (NW) |  |  |  |  |  |  |
| 08-04-2a (2008) | Lijiang(LJ) | *GJ* | A | + | H03 |  |
| 94-183-1a (1994) | Lijiang(LJ) | *GJ* | A | + |  |  |
| 94-183-1b (1994) | Lijiang(LJ) | *GJ* | A | + |  |  |
| 94-184-1a (1994) | Lijiang(LJ) | *GJ* | A | + | H05 |  |
| 94-185-1a (1994) | Lijiang(LJ) | *GJ* | A | + | H05 |  |
| 94-186-1a (1994) | Lijiang(LJ) | *GJ* | A | + | H05 |  |
| 94-187-1a (1994) | Lijiang(LJ) | *GJ* | A | + | H05 |  |
| 94-188-1b (1994) | Lijiang(LJ) | *GJ* | A | + | H05 |  |
| 94-189-1a (1994) | Lijiang(LJ) | *GJ* | A | + | H05 |  |
| 94-194-2a (1994) | Lijiang(LJ) | *GJ* | A | + | H05 |  |
| 94-190-1a (1994) | Lijiang(LJ) | *GJ* | A | - |  |  |
| 94-192-1a (1994) | Lijiang(LJ) | *GJ* | A | - |  |  |
| Northeastern (NE) |  |  |  |  |  |  |
| 97-113-1a (1997) | Zhaotong(ZT) | *GJ* | A | + | H03 |  |
| 97-114-1a (1997) | Zhaotong(ZT) | *GJ* | A | + | H03 |  |
| 97-14-1b (1997) | Zhaotong(ZT) | *GJ* | A | + |  |  |
| 97-16-2a (1997) | Zhaotong(ZT) | *GJ* | A | + |  |  |
| 97-17-1a (1997) | Zhaotong(ZT) | *GJ* | A | + |  |  |
| 97-21-1a (1997) | Zhaotong(ZT) | *GJ* | A | + | H03 |  |
| 97-21-1c (1997) | Zhaotong(ZT) | *GJ* | A | + |  |  |
| 97-21-2a (1997) | Zhaotong(ZT) | *GJ* | A | + |  |  |
| 97-2-1a (1997) | Zhaotong(ZT) | *GJ* | A | + |  |  |
| 97-2-1b (1997) | Zhaotong(ZT) | *GJ* | A | + | H03 |  |
| 97-29-1a (1997) | Zhaotong(ZT) | *GJ* | A | + |  |  |
| 97-37-1a (1997) | Zhaotong(ZT) | *GJ* | A | + | H03 |  |
| 97-4-1a (1997) | Zhaotong(ZT) | *GJ* | A | + | H03 |  |
| 97-42-1a (1997) | Zhaotong(ZT) | *GJ* | A | + |  |  |
| 97-43-1a (1997) | Zhaotong(ZT) | *GJ* | A | + |  |  |
| 97-45-1a (1997) | Zhaotong(ZT) | *GJ* | A | + |  |  |
| 97-46-1a (1997) | Zhaotong(ZT) | *GJ* | A | + |  |  |
| 97-48-1a (1997) | Zhaotong(ZT) | *GJ* | A | + | H03 |  |
| 97-5-2b (1997) | Zhaotong(ZT) | *GJ* | A | + | H04 |  |
| 97-61-1a (1997) | Zhaotong(ZT) | *GJ* | A | + | H05 |  |
| 97-6-1a (1997) | Zhaotong(ZT) | *GJ* | A | + | H03 |  |
| 97-66-1a (1997) | Zhaotong(ZT) | *GJ* | A | + | H03 |  |
| 97-68-1a (1997) | Zhaotong(ZT) | *GJ* | A | + |  |  |
| 97-68-2a (1997) | Zhaotong(ZT) | *GJ* | A | + |  |  |
| 97-69-1a (1997) | Zhaotong(ZT) | *GJ* | A | + | H03 |  |
| 97-70-1a (1997) | Zhaotong(ZT) | *GJ* | A | + |  |  |
| 97-71-1a (1997) | Zhaotong(ZT) | *GJ* | A | + | H03 |  |
| 97-71-2a (1997) | Zhaotong(ZT) | *GJ* | A | + |  |  |
| 97-83-1a (1997) | Zhaotong(ZT) | *GJ* | A | + |  |  |
| 97-83-2a (1997) | Zhaotong(ZT) | *GJ* | A | + | H03 |  |
| 97-83-2b (1997) | Zhaotong(ZT) | *GJ* | A | + |  |  |
| 97-84-1a (1997) | Zhaotong(ZT) | *GJ* | A | + |  |  |
| 97-85-1a (1997) | Zhaotong(ZT) | *GJ* | A | + |  |  |
| 97-85-2a (1997) | Zhaotong(ZT) | *GJ* | A | + |  |  |
| 97-86-1a (1997) | Zhaotong(ZT) | *GJ* | A | + | H03 |  |
| 97-91-1a (1997) | Zhaotong(ZT) | *GJ* | A | + |  |  |
| 97-91-1b (1997) | Zhaotong(ZT) | *GJ* | A | + | H03 |  |
| 97-9-1a (1997) | Zhaotong(ZT) | *GJ* | A | + |  |  |
| 97-9-2a (1997) | Zhaotong(ZT) | *GJ* | A | + | H03 |  |
| YN232 (1995) | Zhaotong(ZT) | *GJ* | A | + | H03 |  |
| 97-16-1a (1997) | Zhaotong(ZT) | *GJ* | V | - |  | "+ + -" |
| 97-17-1a (1997) | Zhaotong(ZT) | *GJ* | V | - |  | "+ - -" |
| 97-18-1b (1997) | Zhaotong(ZT) | *GJ* | V | - |  | "+ - -" |
| 97-47-1a (1997) | Zhaotong(ZT) | *GJ* | V | - |  | "+ - -"^*1^ |
| 97-92-1a (1997) | Zhaotong(ZT) | *GJ* | V | - |  | "+ - -" |
| 97-15-1a (1997) | Zhaotong(ZT) | *GJ* | A | - |  |  |
| 97-18-1a (1997) | Zhaotong(ZT) | *GJ* | A | - |  |  |
| 97-2-2a (1997) | Zhaotong(ZT) | *GJ* | A | - |  |  |
| 97-60-1a (1997) | Zhaotong(ZT) | *GJ* | A | - |  |  |
| 97-73-1a (1997) | Zhaotong(ZT) | *GJ* | A | - |  |  |
| 97-87-1a (1997) | Zhaotong(ZT) | *GJ* | A | - |  |  |
| Southern (S) |  |  |  |  |  |  |
| 34—2 (2008) | Honghe(HH) | *XI* | A | + | H03 |  |
| B14a-2 (2008) | Honghe(HH) | *XI* | A | + |  |  |
| B18a-2-2 (2008) | Honghe(HH) | *XI* | A | + | H03 |  |
| B7-6 (2008) | Honghe(HH) | *XI* | A | + | H02 |  |
| C29 (2008) | Honghe(HH) | *XI* | A | + |  |  |
| J008-3 (2008) | Honghe(HH) | *XI* | A | + | H03 |  |
| J39-1 (2008) | Honghe(HH) | *XI* | A | + | H03 |  |
| J41-1 (2008) | Honghe(HH) | *XI* | A | + | H03 |  |
| YN047 (1995 | Honghe(HH) | *XI* | A | + | H03 |  |
| J01-84 (2008) | Honghe(HH) | *XI* | V | - |  | "- + -" |
| J050-1 (2008) | Honghe(HH) | *XI* | V | - |  | "- - -" |
| 64b-2-1 (2008) | Honghe(HH) | *XI* | A | - |  |  |
| A3 (2008) | Honghe(HH) | *XI* | A | - |  |  |
| A9 (2008) | Honghe(HH) | *XI* | A | - |  |  |
| B18b-2 (2008) | Honghe(HH) | *XI* | A | - |  |  |
| J008-2 (2008) | Honghe(HH) | *XI* | A | - |  |  |
| J57-1 (2008) | Honghe(HH) | *XI* | A | - |  |  |
| 08-39-2d (2008) | Xishuangbanna(BN) | *XI* | V | + | H07 |  |
| 09-03-a (2009) | Xishuangbanna(BN) | *XI* | A | + | H03 |  |
| 09-15-a (2009) | Xishuangbanna(BN) | *XI* | A | + | H03 |  |
| 09-21-a (2009) | Xishuangbanna(BN) | *XI* | A | + | H09 |  |
| 94-19-1a (1994) | Xishuangbanna(BN) | *XI* | V | - |  | "- - -" |
| 94-19-1b (1994) | Xishuangbanna(BN) | *XI* | V | - |  | "- - -" |
| Southwestern (SW) |  |  |  |  |  |  |
| 07-231-2a (2007) | Lincang(LC) | *XI* | V | + | H01 |  |
| 07-229-1a (2007) | Lincang(LC) | *XI* | A | + |  |  |
| 07-239-1a (2007) | Lincang(LC) | *XI* | A | + | H03 |  |
| 07-243-1a (2007) | Lincang(LC) | *XI* | A | + | H03 |  |
| 07-324-2c (2007) | Lincang(LC) | *XI* | A | + | H03 |  |
| YN220 (1995) | Lincang(LC) | *XI* | A | + | H03 |  |
| 07-228-1a (2007) | Lincang(LC) | *XI* | V | - |  | "+ + -" |
| 07-230-1a (2007) | Lincang(LC) | *XI* | V | - |  | "+ - -"^*1^ |
| 07-231-1a (2007) | Lincang(LC) | *XI* | V | - |  | "+ - -"^*1^ |
| 07-240-1a (2007) | Lincang(LC) | *XI* | V | - |  | "+ + -" |
| 07-241-1a (2007) | Lincang(LC) | *XI* | V | - |  | "+ + -" |
| 07-310-1a (2007) | Lincang(LC) | *XI* | V | - |  | "+ - -" |
| 07-311-1a (2007) | Lincang(LC) | *XI* | V | - |  | "+ + -" |
| 07-312-2a (2007) | Lincang(LC) | *XI* | V | - |  | "+ + -" |
| 07-325-1a (2007) | Lincang(LC) | *XI* | V | - |  | "+ - -" |
| 07-241-2a (2007) | Lincang(LC) | *XI* | A | - |  |  |
| 07-309-3a (2007) | Lincang(LC) | *XI* | A | - |  |  |
| 07-324-1b (2007) | Lincang(LC) | *XI* | A | - |  |  |
| 94-64-1b (1994) | Puer(PE) | *XI* | A | + | H06 |  |
| 94-76-1a (1994) | Puer(PE) | *XI* | A | + | H06 |  |
| 94-76-2a (1994) | Puer(PE) | *XI* | A | + | H06 |  |
| 94-77-1a (1994) | Puer(PE) | *XI* | A | + |  |  |
| 95-72-1a (1995) | Puer(PE) | *XI* | A | + |  |  |
| 94-70-1c (1994) | Puer(PE) | *XI* | V | - |  | "+ + -" |
| 95-71-2a (1995) | Puer(PE) | *XI* | V | - |  | "+ - -"^*1^ |
| 95-71-2b (1995) | Puer(PE) | *XI* | V | - |  | "+ - -"^*1^ |
| Southestern (SE) |  |  |  |  |  |  |
| 07-168-1a (2007) | Wenshan(WS) | *XI* | A | + | H05 |  |
| 07-175-1a (2007) | Wenshan(WS) | *XI* | A | + |  |  |
| 07-176-1a (2007) | Wenshan(WS) | *XI* | A | + |  |  |
| 07-177-1a (2007) | Wenshan(WS) | *XI* | A | + |  |  |
| 07-183-1a (2007) | Wenshan(WS) | *XI* | A | + |  |  |
| 07-186-1a (2007) | Wenshan(WS) | *XI* | A | + | H05 |  |
| 95-69-2a (1995) | Wenshan(WS) | *XI* | A | + |  |  |
| 95-69-2b (1995) | Wenshan(WS) | *XI* | A | + | H03 |  |
| 95-70-1a (1995) | Wenshan(WS) | *XI* | A | + | H08 |  |
| 07-119-1a (2007) | Wenshan(WS) | *XI* | V | - |  | "+ - -" |
| 07-113-2a (2007) | Wenshan(WS) | *XI* | A | - |  |  |
| 07-174-1a (2007) | Wenshan(WS) | *XI* | A | - |  |  |
| 07-177-2c (2007) | Wenshan(WS) | *XI* | A | - |  |  |
| 95-69-1a (1995) | Wenshan(WS) | *XI* | A | - |  |  |

^a^ *GJ* and *XI* indicate isolates from *Geng/Japonica* and *Xian/Indica* rice production area, respectively; ^*1^the middle amplified fragment was not target fragment, homologous to part sequence of cellobiose dehydrogenase of *Magnaporthe oryzae*; ^*2^ the middle fragment was not target fragment, homologous to part sequence of *Enterobacter cloacae*.
