# Supplementary material for: Novel Variation and Evolution of AvrPiz-t of Magnaporthe oryzae in Field Isolates
Source: Front Genet. 2020 Aug 28;11:746. doi: 10.3389/fgene.2020.00746 (PMC7484972; doi:10.3389/fgene.2020.00746)
Supplement: Supplementary file 3 [file Table_2.docx]

**Table S2.** Primers used in this study

| **Primer name (abbr.)** | **sequences (5'-3')** | **Location^a^** | **TM** | **Usage** | **reference** |
| --- | --- | --- | --- | --- | --- |
| AvztF (F) | TTTAAATTAAGCTTCCAACGTAGAGCGTATGC | 1～18^b^ | 54.2 | *AvrPiz-t* amplification | Published [23] |
| AvztR (R) | AAATTTGAGCTCTCCGTTGAAGCAACAGGATC | 2488～2507^c^ | 56.7 | *AvrPiz-t* amplification | Published [23] |
| AvrPizt-F1 (F1) | GCTGACCATTGGAATGCGAG | -161～-140 | 57.4 | The 5′fragment amplification | This study |
| AvrPizt-R1 (R1) | GTTTGACTCGTCCCCAGGTT | 618～637 | 57.4 | The 5′fragment amplification and internal sequence | This study |
| AvrPizt-F2 (F2) | CATCAAGCATACCAACGGGC | 472～491 | 57.4 | Middle fragment amplification | This study |
| AvrPizt-R2 (R2) | TGACGGGATTTGGTTCGACT | 1424～1443 | 55.4 | Middle fragment amplification and internal sequence | This study |
| AvrPizt-F3 (F3) | CCTTCTCCACCATCCAGCCAT | 1222～1242 | 59.5 | 3′ fragment amplification and sequencing | This study |
| AvrPizt-R3 (R3) | GGTTATGATGCCAGGAGCCAG | 2735～2755 | 59.5 | 3′ fragment amplification | This study |
| C8-W1F-F01 (F01) | AAATTCAATTAGGAACGAGCCAC | 1994～2016 | 54.2 | internal sequence | This study |
| F3-W1F-F09 (F09) | ATAAGGAAGAAGGCGGGTTG | 1668～1687 | 55.4 | internal sequence | This study |
| C8-CW1F-B08 (B08) | TTCAATAAACCCTTCAGCCAA | 2091-2111 | 51.7 | internal sequence | This study |

^a^: Primer’s location in the *AvrPiz-t* gene (EU837058). Negeative value representative the primer located in the gene’s upstream, more than 2507 showed that the primer located in the downstream of the complete gene. ^b^ and ^c^ , location of shade sequence of primer AvztF, AvztR respectively.
