# Supplementary material for: Novel Variation and Evolution of AvrPiz-t of Magnaporthe oryzae in Field Isolates
Source: Front Genet. 2020 Aug 28;11:746. doi: 10.3389/fgene.2020.00746 (PMC7484972; doi:10.3389/fgene.2020.00746)
Supplement: Supplementary file 4 [file Table_3.docx]

**Table S3.** Results of nested clade analyses of the geographical distance of *AvrPiz-t* haplotypes of *Magnaporthea oryzae*

| Haplotype and Clade | Type of geographical distance | | chi-square statistics | p |
| --- | --- | --- | --- | --- |
|  | Within clade (Dc) | Nested clade (Dn) |  |  |
| **Clade 1-3** |  |  | 134.42^*^ | 0.0004 |
| H9 T | 0 | 285.65 |  |  |
| H6 T | 0.0^*s^ | 166.68 |  |  |
| H8 T | 0 | 315.42 |  |  |
| H3 I | 226.2225 | 227.88 |  |  |
| I-T | 226.2225^*l^ | 7.66 |  |  |
| **Clade 1-5** |  |  | 32.45 | 0.095 |
| H2 I | 0 | 247.63 |  |  |
| H5 I | 180.71 | 180.79 |  |  |
| H4 I | 0 | 280.55 |  |  |
| **Clade 2-2** |  |  | 73.60^*^ | 0.004 |
| 1-2 T | 0 | 121.48 |  |  |
| 1-4 T | 0 | 305.35 |  |  |
| 1-5 T | 185.19 | 197.67 |  |  |
| 1-3 I | 226.92 | 228.33 |  |  |
| I-T | 63.95^*l^ | 32.45 |  |  |
| **Total Cladogram** |  |  | 19.19 | 0.24 |
| 2-1 T | 0 | 173.18 |  |  |
| 2-2 T | 220.89 | 221.04 |  |  |

An “s” superscript indicates significantly smaller than random pattern at 5%; an “l” superscript indicates significantly larger than random pattern at 5%.
