# Supplementary material for: Novel Variation and Evolution of AvrPiz-t of Magnaporthe oryzae in Field Isolates
Source: Front Genet. 2020 Aug 28;11:746. doi: 10.3389/fgene.2020.00746 (PMC7484972; doi:10.3389/fgene.2020.00746)
Supplement: Supplementary file 5 [file Table_4.docx]

**Table S4.** Past events inferred from the nested clade phylogeographical analysis inference keys, which possibly shape the present distribution pattern of the genetic structure of *Magnaporthea oryzae*.

| Clade | Chain of inference | inferences |
| --- | --- | --- |
| Clade 1-3 | 1-2-3-4 NO | restricted dispersal by distance |
| Clade 1-5 | Null hypothesis cannot be rejected. |  |
| Clade 2-2 | 1-2-3-4 NO | restricted dispersal by distance |
| Total Cladogram | Null hypothesis cannot be rejected. |  |
